# Correction: Decoding reveals the neural representation of perceived and imagined musical sounds

**DOI:** 10.1371/journal.pbio.3003445

**Published:** 2025-10-16

**Authors:** David R. Quiroga-Martinez, Gemma Fernández Rubio, Leonardo Bonetti, Kriti G. Achyutuni, Athina Tzovara, Robert T. Knight, Peter Vuust

## Notice of Republication

There is an error in the second author’s name. The correct name is: Gemma Fernández Rubio. The correct citation is: Quiroga-Martinez DR, Fernández Rubio G, Bonetti L, Achyutuni KG, Tzovara A, Knight RT, et al. (2024) Decoding reveals the neural representation of perceived and imagined musical sounds. PLoS Biol 22(10): e3002858. https://doi.org/10.1371/journal.pbio.3002858
