# Supplementary material for: Decoding reveals the neural representation of perceived and imagined musical sounds
Source: PLoS Biol. 2024 Oct 21;22(10):e3002858. doi: 10.1371/journal.pbio.3002858 (PMC11527242; doi:10.1371/journal.pbio.3002858)
Supplement: S1 Appendix — (PDF) [file pbio.3002858.s001.pdf]

S1 Appendix

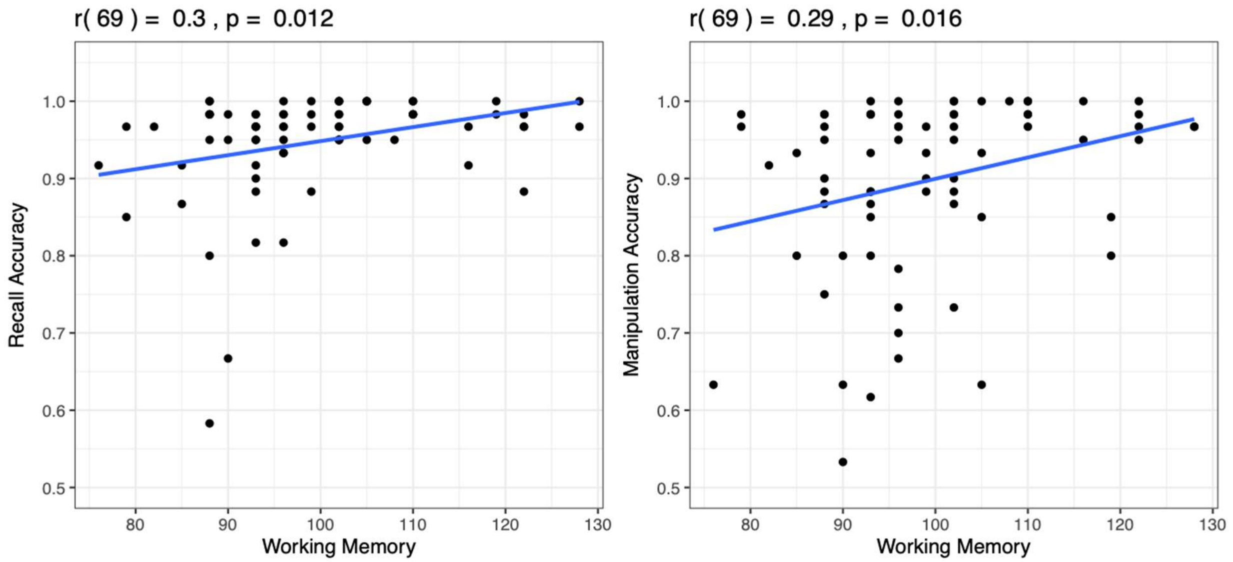

Fig A. The relationship between working memory and recall and manipulation accuracy. We report Pearson correlations between working memory scores, calculated using the Wechsler Adult Intelligence Scale (WAIS), and recall and manipulation accuracy scores for each subject. Results show a positive correlation between working memory and both recall and manipulation accuracy. This relationship suggests that general working memory skills are beneficial for the retention and manipulation of (non-verbal) auditory objects in the context of an active imagery task. See supporting file **S1 Fig** for data related to the current figure.

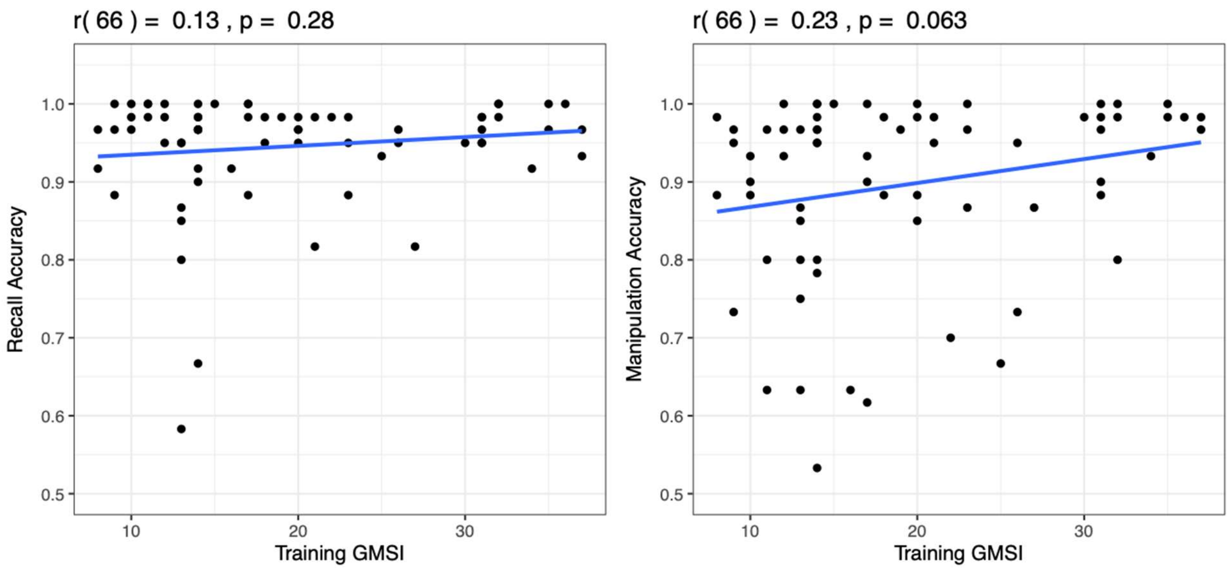

Fig B. The relationship between music training (training subscale from the Goldsmiths Musical Sophistication Index - GMSI) and recall and manipulation accuracy. We report Pearson correlations between training GMSI scores, and recall and manipulation accuracy scores for each participant. Results show small positive correlations between music training and both recall and manipulation accuracy which, nonetheless, were statistically non-significant. See supporting file **S1 Fig** for data related to the current figure.

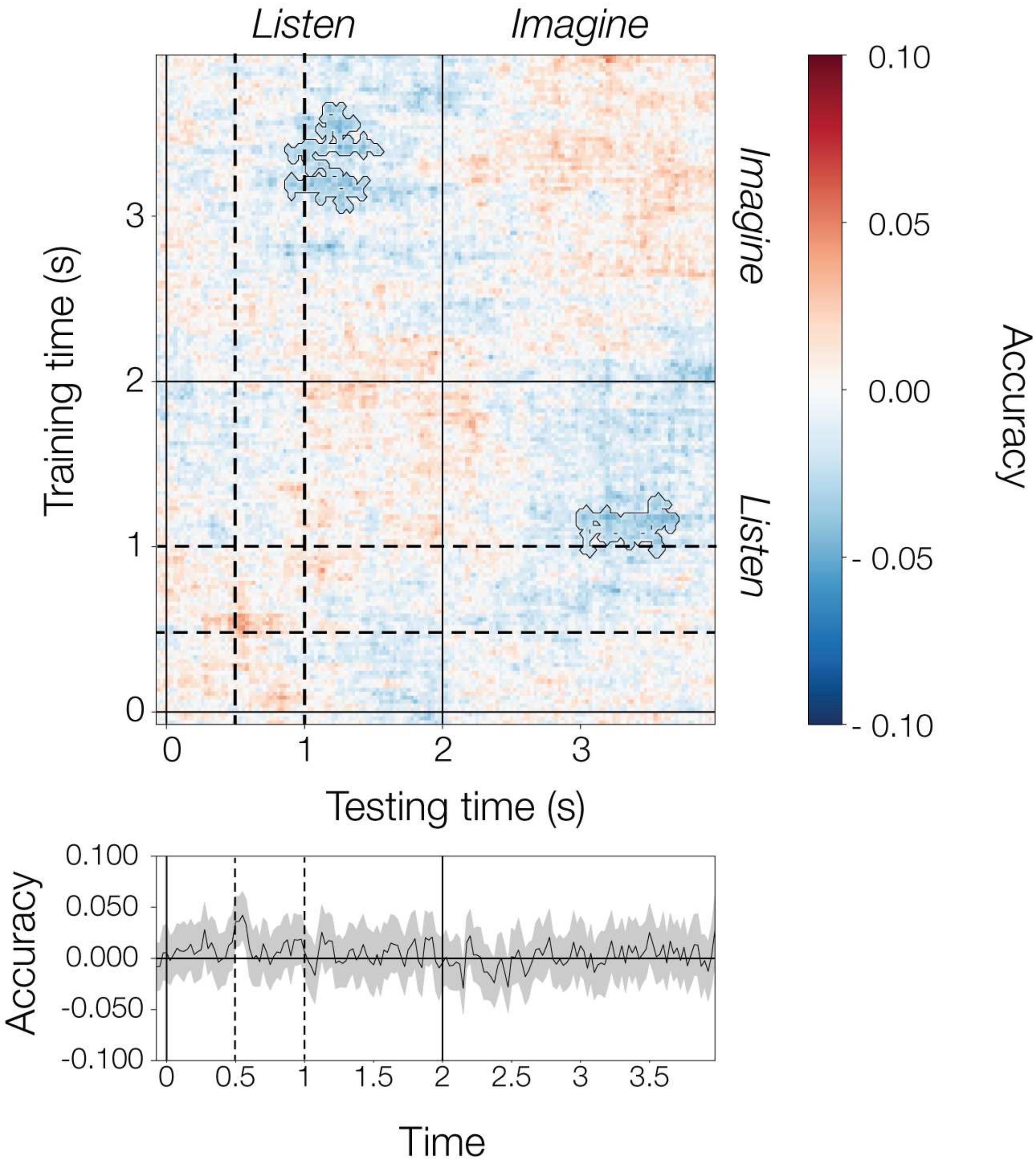

Fig C. Difference between manipulation and recall for within-condition testing. Note how significant below-chance accuracy (contours) emerges when training on listening and testing on imagination and vice-versa. Bottom time-course depicts accuracy differences at the diagonal. Dashed lines mark the onset of the second (0.5s) and third (1s) sounds of the melodies. See the online repository for the data, statistical output and analysis scripts related to the current figure.

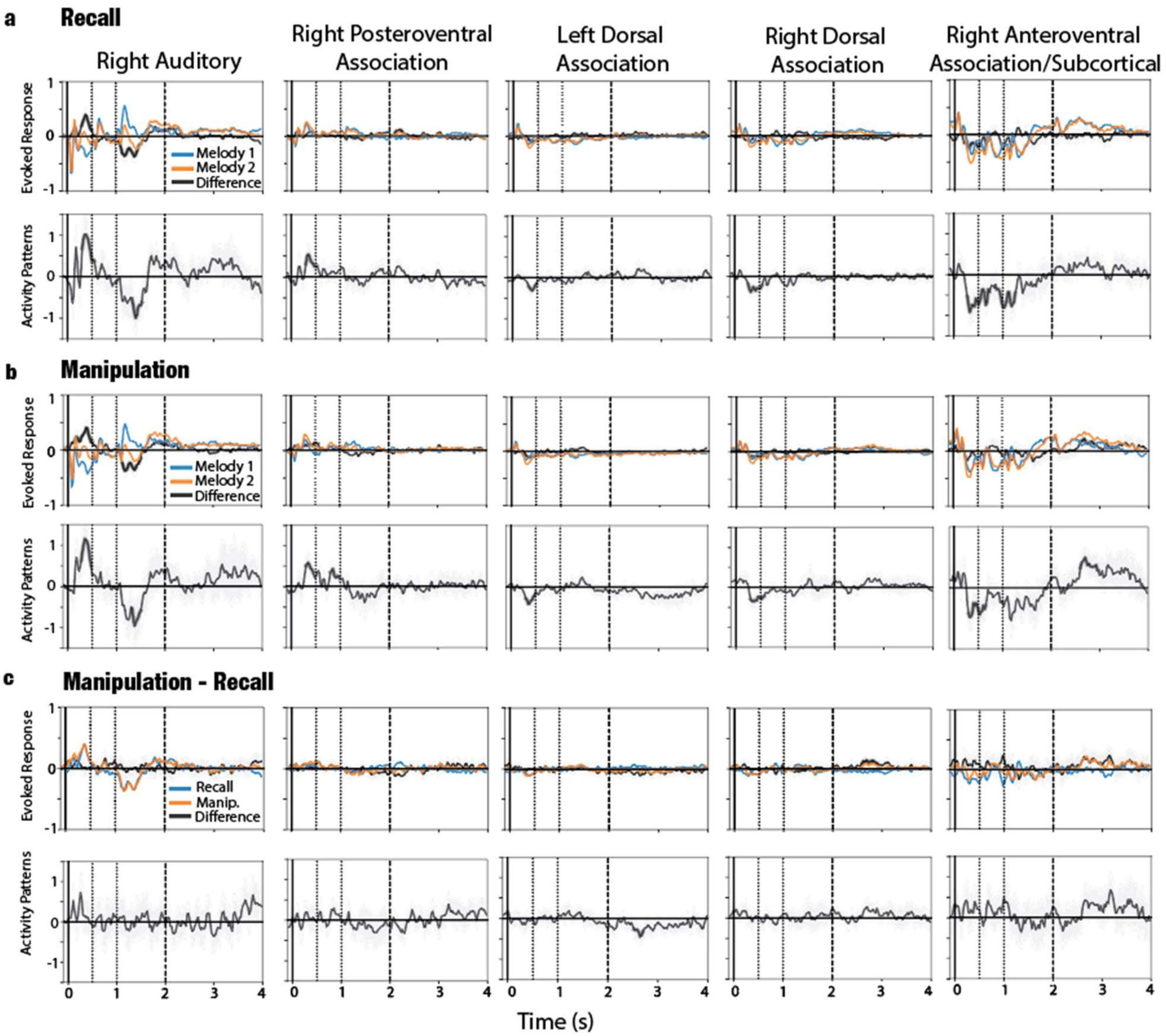

Fig D. Time course of source-level evoked responses and decoding patterns for five different regions of interest, according to the Desikan-Killiany parcellation (1): ***Right Auditory*** (ctx-rh-superiortemporal, ctx-rh-bankssts, ctx-rh-transversetemporal), ***Right Posteroventral Association*** (ctx-rh-fusiform, ctx-rh-inferiortemporal, Right-Hippocampus, ctx-rh-parahippocampal', ctx-rh-isthmuscingulate, ctx-rh-precuneus,ctx-rh-lingual), ***Left Dorsal Association*** (ctx-lh-rostralmiddlefrontal, ctx-lh-superiorfrontal, ctx-lh-parstriangularis, ctx-lh-parsopercularis, ctx-lh-parsorbitalis, ctx-lh-insula), ***Right Dorsal Association*** (ctx-rh-rostralmiddlefrontal, ctx-rh-superiorfrontal, ctx-rh-parstriangularis, ctx-rh-parsopercularis, ctx-rh-parsorbitalis, ctx-rh-insula), and ***Right Anteroventral Association/Subcortical*** (ctx-rh-medialorbitofrontal, ctx-rh-lateralorbitofrontal, Right-Accumbens-area, Right-Caudate, Right-Putamen, Right-Pallidum, Right-Thalamus-Proper, ctx-rh-rostralanteriorcingulate). Bold segments indicate significant periods.

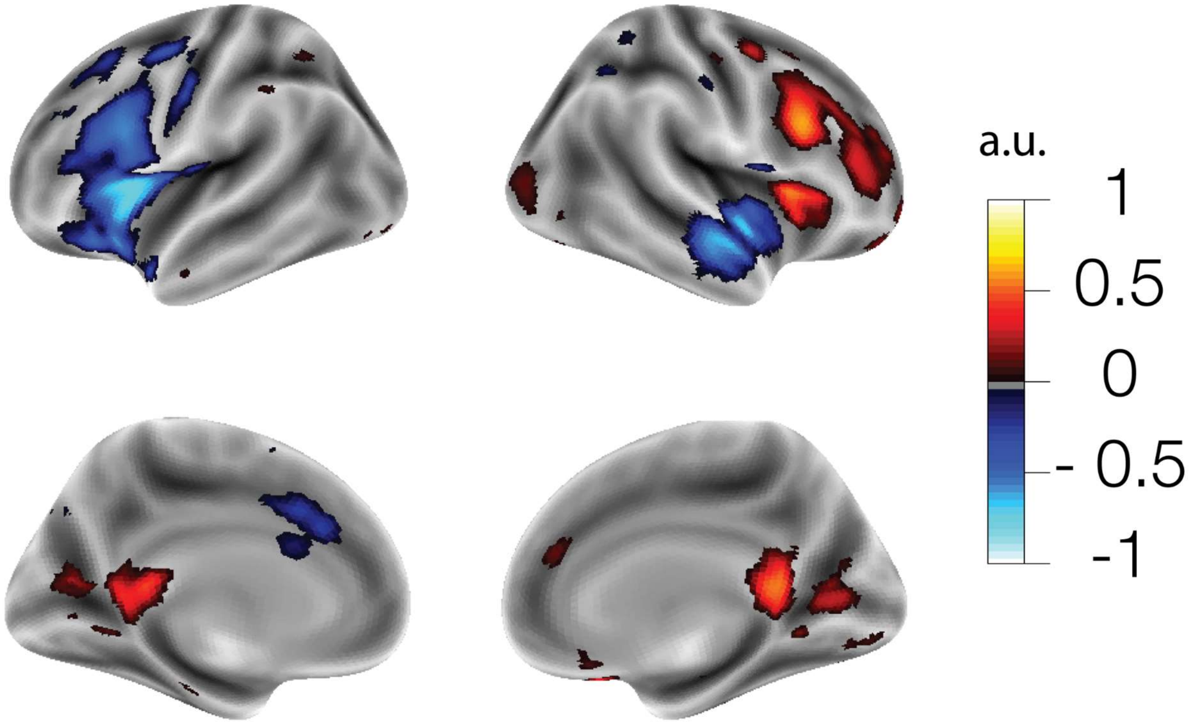

Fig E. Uncorrected (t > 1.99) significant differences between manipulation and recall for the imagination period (2s - 4s). Note the bilateral frontal and medial parietal differences.

Exploratory analysis: Relationship of neural decoding accuracy with behavioral measures For completeness, in exploratory analyses, we tested whether neural decoding accuracy was related to behavioral accuracy, musical training or vividness ratings. To this purpose, we took the mean of neural decoding accuracy scores in the generalization matrices (**Fig 2**) for the listening (0s - 2s) and imagination (2s - 4s) periods separately (i.e., the left-bottom and top-right quadrants). Since below-chance and above-chance accuracy values were present, we derived an unsigned neural discrimination measure (**ND**) by subtracting 0.5 (i.e., the chance level) and computing the absolute value before taking the mean of the corresponding period. ND represents the information in the neural signal that drives above or below-chance decoding. In the manipulation condition, ND was significantly correlated with behavioral accuracy for the listening period (r = .24, p = .045) and marginally for the imagination period (r = .22, p = .052), before multiple comparisons corrections (**Table A** below). No other significant associations were found.

Table A. Pearson's correlation coefficients (***r***), and corresponding *p*-values with (p_{FDR}) and without (***p***) false-discovery-rate correction, between neural discrimination (**ND**) and three variables of interest: Behavioral accuracy, vividness ratings and music training (GMSI scores).

Condition / Period	Behavioral Accuracy			Vividness Ratings			Music Training (GMSI)		
	r	*p*	p_{FDR}	*r*	*p*	p_{FDR}	*r*	*p*	p_{FDR}
Recall / listening	-.07	.553	.885	.08	.523	.885	0	.989	.989
Recall / imagination	-.15	.224	.674	.16	.198	.673	.06	.639	.885
Manipulation / listening	**.24**	**.045**	.315	-.01	.924	.989	.1	.425	.885
Manipulation / imagination	.23	.052	.315	-.04	.763	.916	.05	.664	.885

In addition, we explored whether the likelihood of a correct answer in the task (and corresponding response times), was related to the likelihood of correct neural decoding in the listening and imagination periods, at the single-trial level. We used logistic mixed-effects models (R, lme4) (2, 3) to evaluate whether participants' target melody identification predicted correct melody discrimination from neural activity at each time point of the trial. We did this analysis separately for the recall and manipulation blocks, and for the listening and imagination periods. We included participant as random effect for both intercept and slopes. We obtained *p*-values for the relevant coefficients using the Satterthwaite approximation of degrees of freedom (4). The same analysis was done to predict neural accuracy from response times, as measured from the beginning of the test melody. No significant relationships were detected in these analyses, likely due to the ceiling effect in behavioral performance (**Fig. F** and **G** below).

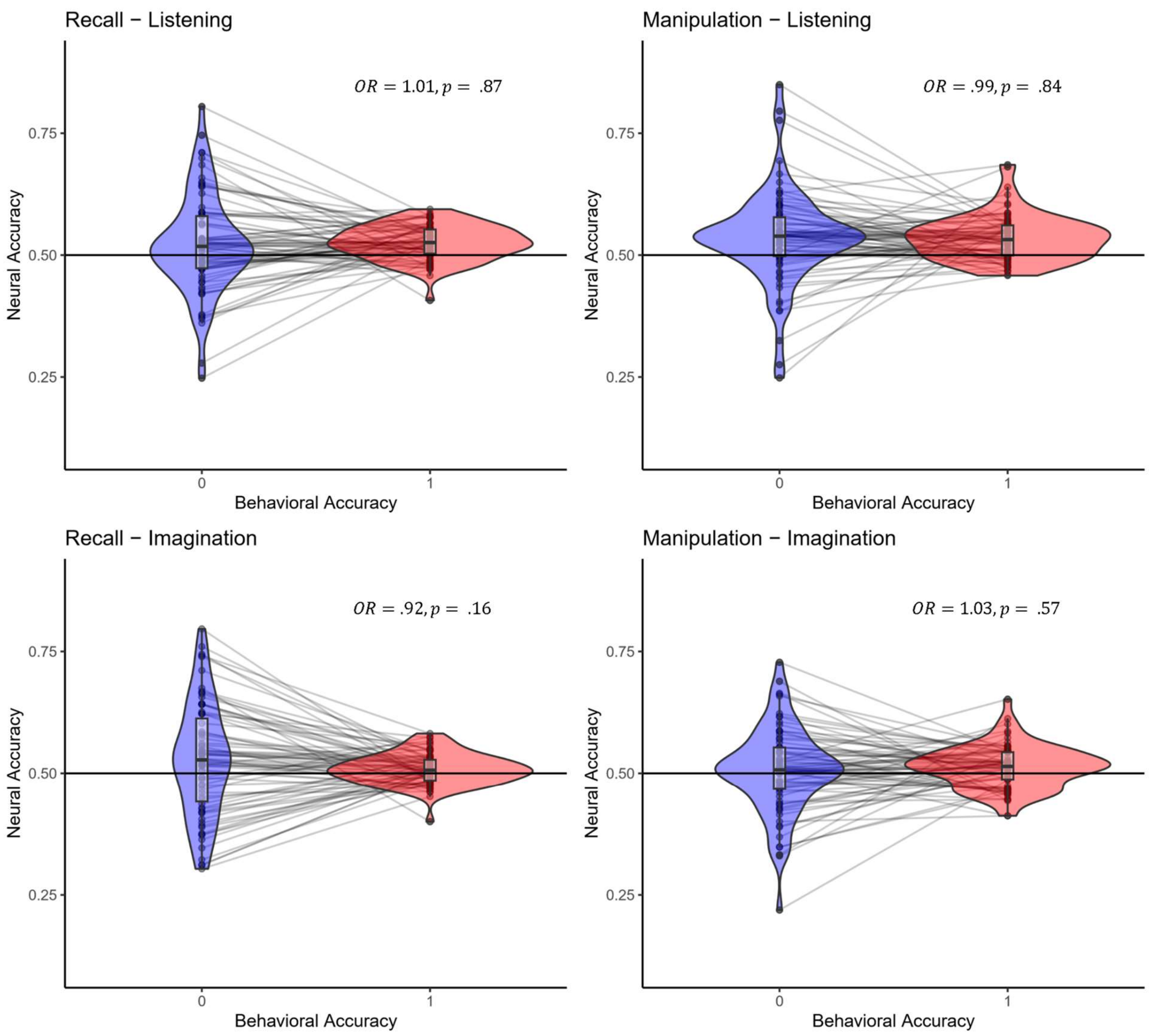

Fig F. Relationship between neural decoding accuracy and behavioral performance at the single trial level. Using logistic regression (i.e., generalized mixed-effects models) we evaluated how well behavioral accuracy (correct=1, incorrect=0) predicted neural decoding of melody identity across all trials, participants and timepoints for the two conditions (recall, manipulation) and two different periods (listening, imagination) separately. The models included participant as a random effect (both for intercept and slopes). Each dot represents a prediction from the model for a single subject. Boxplots depict the median and interquartile range. The larger variance for incorrect trials reflects their smaller number compared to correct trials. Note that some participants did not exhibit any incorrect trials, but the model still extrapolated neural accuracy coefficients for them. See supporting file **S5 Fig** for data related to the current figure.

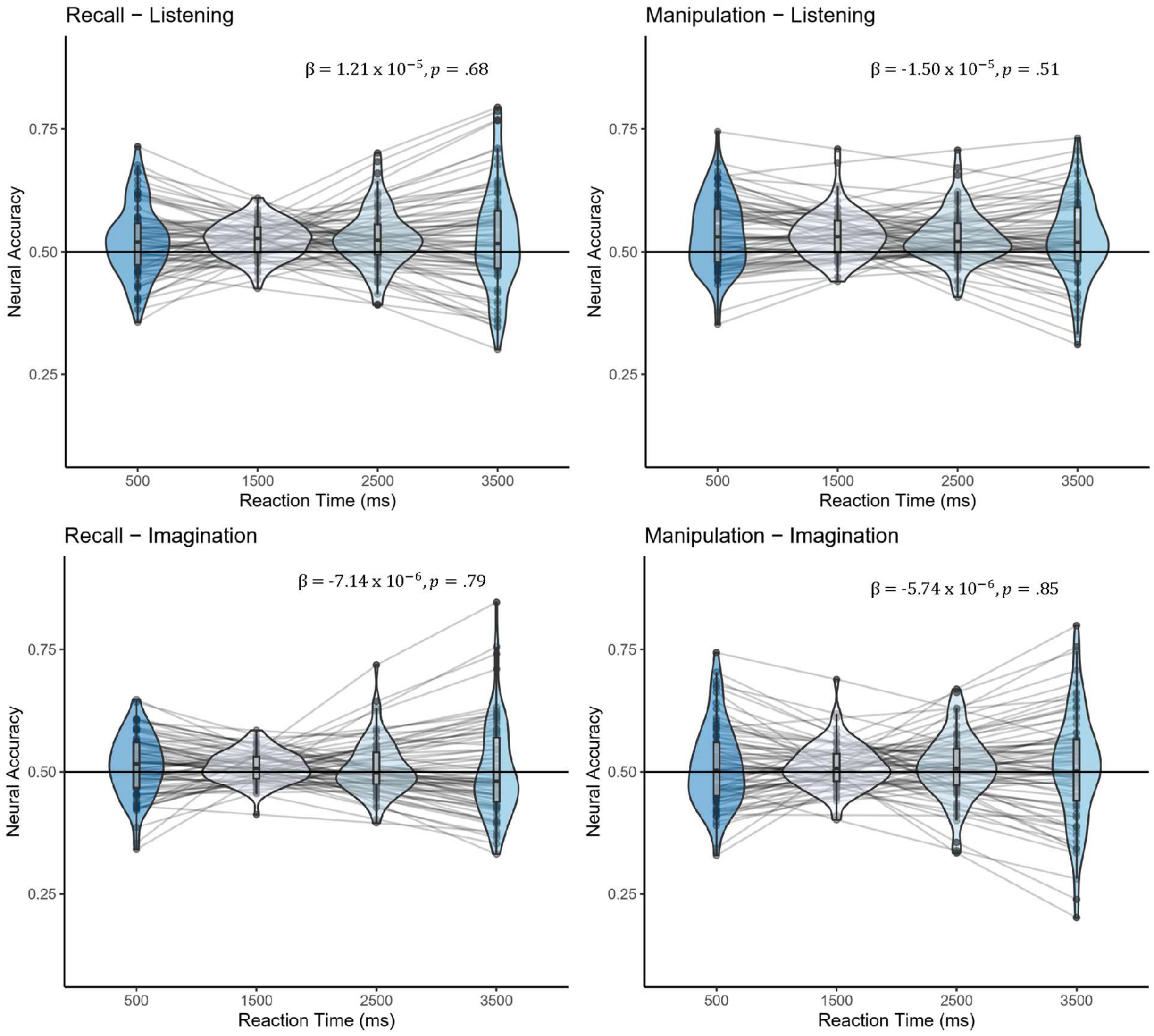

Fig G. Relationship between neural decoding accuracy and reaction time at the single trial level. Using logistic regression (i.e., generalized mixed-effects models), we evaluated how well reaction time predicted neural decoding of melody identity across all trials, participants and time points for the two conditions (recall, manipulation) and two different periods (listening, imagination) separately. The models included participant as a random effect (both for intercept and slope). Each dot represents a prediction from the model for a single subject at different RTs (500ms, 1500ms, 2500ms, 3500ms). Boxplots depict the median and interquartile range. See supporting file **S6 Fig** for data related to the current figure.

Fig H. Source localization of decoding patterns using inverse operators estimated with the data covariance of the listening (sounds 1, 2, and 3) and imagination period separately. Significant clusters are displayed.

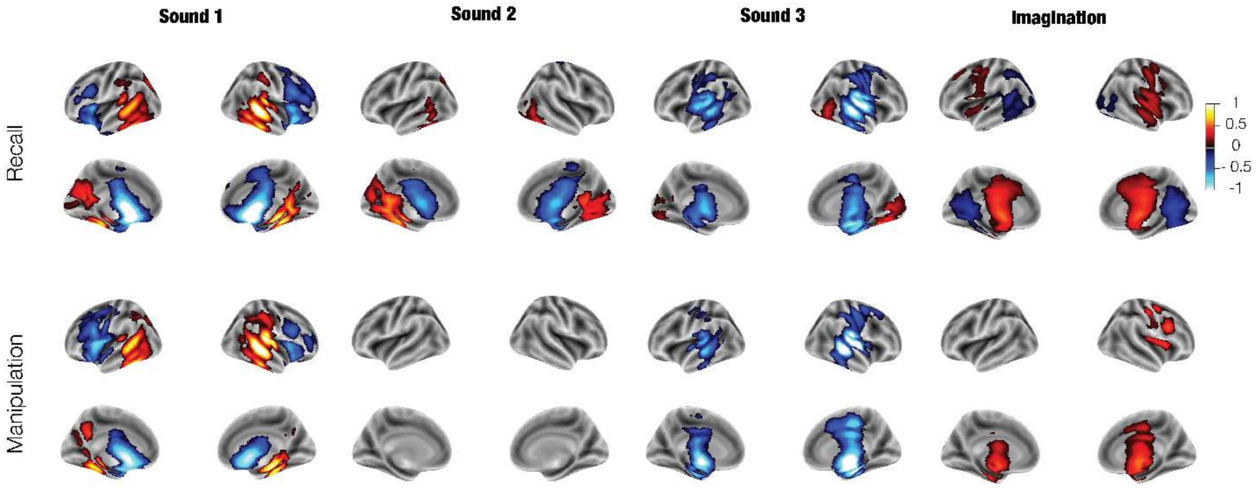

Table B. Summary of all significant clusters found for the tests performed on time-generalized accuracy matrices.

condition	training peak time (s)	testing peak time (s)	cluster p-value	peak t-value	peak accuracy
train on recall and test on recall	0.2	0.2	0	7.13	0.573
	1.23	1.23	0	7.949	0.589
	2.33	2.28	0	6.863	0.547
	3.15	3.1	0	5.555	0.543
	1.38	0.53	0.007	-2.185	0.485
	0.18	1.42	0.008	-2.109	0.486
train on manipulation and test on manipulation	1.2	1.2	0	9.675	0.602
	3.35	3.65	0	4.542	0.549
	1.18	0.5	0.016	-2.019	0.486
	0.53	1.9	0.014	-2.017	0.485
train on recall and test on manipulation	0.2	0.18	0	7.767	0.569
	1.2	1.2	0	7.927	0.577
	1.4	0.18	0	-2.174	0.488
	0.08	1.42	0	-2.069	0.486
	3.18	2.78	0.048	-2.061	0.485
	3.3	2.98	0.011	-2.018	0.485
	3.35	3.78	0.034	-2.01	0.484
train on manipulation and test on recall	0.18	0.18	0	9.901	0.58
	1.2	1.2	0	8.008	0.574
	1.5	0.35	0	-2.06	0.487
	0.38	1.63	0	-2.047	0.485
	2.9	2.92	0.011	-2.056	0.487
	3.28	3.52	0.018	-2.039	0.485
recall minus manipulation (within-condition testing)	3.2	1.33	0.033	-4.036	-0.049
	3.42	1.25	0.016	-5.289	-0.053
	1.18	3.2	0.009	-4.625	-0.05
test on recall (between minus within)	2.3	2.28	0.03	-5.762	-0.058

	3.38	3.45	0	-5.563	-0.067
test on manipulation (between minus within)	1.88	1.9	0.035	-4.545	-0.064
	3.22	3.32	0	-4.434	-0.079

Table C. Summary of all significant clusters found for the tests performed on patterns of activation at the source level. Coordinates of peak activity (x, y, z) are given in standard MNI space.

period or comparison	condition	cluster No	x	y	z	cluster p-value	peak t-value	peak activation
first sound	recall	1	50	-40	-15	0.0002	6.19	0.91
		2	0	5	-5	0.0002	-7.67	-1.45
	manipulation	1	-65	-55	0	0.0066	5.16	0.23
		2	50	-40	-15	0.0048	5.31	0.83
		3	-20	5	15	0.0002	-5.72	-1.04
second sound	recall	1	-30	-40	-25	0.0012	4.73	0.72
		2	0	-5	10	0.0046	-4.98	-0.88
	manipulation	1	-35	-10	50	0.0014	-4.61	-0.42
third sound	recall	1	25	-75	-15	0.0214	4.23	0.39
		2	0	-15	5	0.0002	-5.00	-0.82
	manipulation	1	25	-15	-5	0.0002	-5.28	-1.61
imagination	recall	1	0	-10	25	0.0002	5.28	0.42
		2	15	-60	15	0.0008	-4.67	-0.31
	manipulation	1	5	-10	5	0.0008	4.64	0.48
	difference	1	-25	15	30	0.0328	-3.53	-0.48
third minus first sound	recall	1	0	25	-20	0.0004	5.67	1.12
		2	50	-35	-20	0.003	-6.35	-1.56
		3	-55	-40	-5	0.0172	-4.78	-1.23
	manipulation	1	-25	20	15	0.0038	4.16	0.87
		2	55	-35	-5	0.0002	-5.96	-2.14
imagination minus listening	recall	1	-5	-10	5	0.0002	8.23	1.14
		2	-5	-65	25	0.0002	-6.5	-0.44
	manipulation	1	0	-10	5	0.0002	6.64	0.93

Table D. Peak t-value for each region in the **right** hemisphere that overlapped with significant clusters in each of the tested periods and conditions. The Desikan-Killiany parcellation was used to obtain anatomical labels.

Period/comparison	Condition	Cluster No	Label	t-value
First sound	recall	1	ctx-rh-inferiortemporal	6.14
			ctx-rh-fusiform	6.11
			ctx-rh-middletemporal	5.32
			ctx-rh-bankssts	5.29
			ctx-rh-superiortemporal	4.66
			ctx-rh-parahippocampal	4.46
			Right-Hippocampus	4.16
			ctx-rh-inferiorparietal	3.42
			ctx-rh-isthmuscingulate	3.35
			ctx-rh-lingual	3.32
			ctx-rh-transversetemporal	3.16
			ctx-rh-supramarginal	2.99
			ctx-rh-insula	2.78
			ctx-rh-precuneus	2.73
			Right-Thalamus-Proper	2.61
			ctx-rh-postcentral	2.58
		2	Right-Accumbens-area	-6.79
			ctx-rh-medialorbitofrontal	-6.47
			Right-Caudate	-6.26
			Right-Thalamus-Proper	-6.21

			ctx-rh-rostralanteriorcingulate	-5.8
			Right-Putamen	-5
			ctx-rh-lateralorbitofrontal	-4.63
			Right-Pallidum	-4.62
			ctx-rh-rostralmiddlefrontal	-4.3
			ctx-rh-insula	-4.26
			ctx-rh-parstriangularis	-4.01
			ctx-rh-parsopercularis	-3.88
			ctx-rh-parsorbitalis	-3.79
			Right-Amygdala	-3.56
			ctx-rh-caudalmiddlefrontal	-3.47
			ctx-rh-posteriorcingulate	-3.37
			ctx-rh-paracentral	-3.29
			ctx-rh-superiorfrontal	-3.29
			ctx-rh-superiortemporal	-3.14
			ctx-rh-entorhinal	-2.96
			ctx-rh-temporalpole	-2.77
			ctx-rh-precentral	-2.6
			Right-Hippocampus	-2.57
			ctx-rh-caudalanteriorcingulate	-2.54
	manipulation	2	ctx-rh-middletemporal	5.23
			ctx-rh-inferiortemporal	5.09
			ctx-rh-bankssts	4.95

			ctx-rh-superiortemporal	4.71
			ctx-rh-supramarginal	4.49
			ctx-rh-inferiorparietal	4.07
			ctx-rh-postcentral	4.02
			ctx-rh-fusiform	3.91
			ctx-rh-transversetemporal	3.87
			ctx-rh-parahippocampal	3.83
			Right-Hippocampus	3.8
			ctx-rh-insula	3.58
			ctx-rh-lingual	2.71
			Right-Thalamus-Proper	2.7
			ctx-rh-isthmuscingulate	2.5
			ctx-rh-entorhinal	2.39
			Right-Putamen	2.27
		3	Right-Thalamus-Proper	-4.72
			Right-Accumbens-area	-4.65
			Right-Caudate	-4.38
			ctx-rh-medialorbitofrontal	-4.27
			ctx-rh-rostralanteriorcingulate	-3.78
			ctx-rh-superiorfrontal	-3.76
			ctx-rh-precentral	-3.48
			ctx-rh-insula	-3.42
			ctx-rh-lateralorbitofrontal	-3.37

			Right-Putamen	-3.34
			ctx-rh-rostralmiddlefrontal	-3.3
			ctx-rh-parstriangularis	-3.23
			ctx-rh-parsopercularis	-3.19
			ctx-rh-parsorbitalis	-2.96
			ctx-rh-caudalanteriorcingulate	-2.95
			Right-Pallidum	-2.95
			ctx-rh-caudalmiddlefrontal	-2.77
			ctx-rh-superiortemporal	-2.31
			ctx-lh-entorhinal	-2.3
Second sound	recall	1	ctx-rh-isthmuscingulate	3.72
			ctx-rh-lingual	3.6
			ctx-rh-precuneus	3.54
			ctx-rh-fusiform	3.22
			ctx-rh-lateraloccipital	3.17
			ctx-rh-pericalcarine	3.14
			ctx-rh-cuneus	2.8
			ctx-rh-inferiortemporal	2.65
			ctx-rh-inferiorparietal	2.57
			ctx-rh-parahippocampal	2.54
			Right-Hippocampus	2.35
		2	Right-Thalamus-Proper	-4.91
			Right-Caudate	-4.25
			Right-Amygdala	-3.73

			Right-Accumbens-area	-3.63
			ctx-rh-posteriorcingulate	-3.59
			Right-Putamen	-3.51
			Right-Pallidum	-3.51
			Right-Hippocampus	-3.33
			ctx-rh-entorhinal	-3.14
			ctx-rh-medialorbitofrontal	-3.01
			ctx-rh-lateralorbitofrontal	-2.8
			ctx-rh-paracentral	-2.71
			ctx-rh-rostralanteriorcingulate	-2.55
			ctx-rh-precentral	-2.53
			ctx-rh-precuneus	-2.51
			ctx-rh-caudalanteriorcingulate	-2.41
			ctx-rh-caudalmiddlefrontal	-2.35
			ctx-rh-insula	-2.15
			ctx-rh-isthmuscingulate	-2.03
	manipulation	1	ctx-rh-precentral	-3.96
			Right-Thalamus-Proper	-3.55
			ctx-rh-caudalmiddlefrontal	-3.35
			ctx-rh-parsopercularis	-3.27
			ctx-rh-insula	-3.1
			ctx-rh-rostralmiddlefrontal	-3.04
			Right-Putamen	-3.04

			ctx-rh-posteriorcingulate	-3.03
			ctx-rh-postcentral	-2.78
			ctx-rh-superiorfrontal	-2.74
			ctx-rh-caudalanteriorcingulate	-2.68
			ctx-rh-paracentral	-2.57
			Right-Pallidum	-2.4
			Right-Caudate	-2.37
			ctx-rh-supramarginal	-2.25
Third sound	recall	1	ctx-rh-fusiform	3.81
			ctx-rh-lingual	3.73
			ctx-rh-pericalcarine	3.69
			ctx-rh-lateraloccipital	3.63
			ctx-rh-cuneus	3.62
			ctx-rh-middletemporal	2.67
			ctx-rh-inferiortemporal	2.46
			ctx-rh-inferiorparietal	2.33
		2	Right-Thalamus-Proper	-4.94
			ctx-rh-superiortemporal	-4.78
			ctx-rh-inferiortemporal	-4.69
			ctx-rh-postcentral	-4.59
			ctx-rh-middletemporal	-4.53
			Right-Pallidum	-4.49
			ctx-rh-supramarginal	-4.46
			ctx-rh-bankssts	-4.34

			ctx-rh-transversetemporal	-4.31
			ctx-rh-fusiform	-4.28
			Right-Putamen	-4.23
			Right-Amygdala	-4.13
			Right-Hippocampus	-4.07
			ctx-rh-precentral	-3.84
			ctx-rh-insula	-3.82
			ctx-rh-entorhinal	-3.76
			ctx-rh-parahippocampal	-3.59
			Right-Caudate	-3.24
			ctx-rh-posteriorcingulate	-2.92
			ctx-rh-paracentral	-2.61
			ctx-rh-superiorfrontal	-2.57
			ctx-rh-caudalmiddlefrontal	-2.52
			ctx-rh-parsopercularis	-2.45
			Right-Accumbens-area	-2.29
			ctx-rh-rostralmiddlefrontal	-2.01
	manipulation	1	Right-Pallidum	-5.28
			Right-Putamen	-5.22
			Right-Thalamus-Proper	-4.76
			ctx-rh-precentral	-4.71
			Right-Caudate	-4.6
			ctx-rh-supramarginal	-4.58

			Right-Amygdala	-4.51
			Right-Hippocampus	-4.44
			ctx-rh-postcentral	-4.43
			ctx-rh-middletemporal	-4.36
			ctx-rh-posteriorcingulate	-4.32
			ctx-rh-superiortemporal	-4.28
			ctx-rh-paracentral	-4.08
			ctx-rh-bankssts	-4.03
			ctx-rh-insula	-3.9
			ctx-rh-superiorfrontal	-3.84
			ctx-rh-transversetemporal	-3.6
			ctx-rh-caudalanteriorcingulate	-3.38
			ctx-rh-caudalmiddlefrontal	-3.36
			ctx-rh-entorhinal	-3.07
			ctx-rh-parahippocampal	-3.07
			ctx-rh-fusiform	-2.92
			ctx-rh-isthmuscingulate	-2.9
			ctx-rh-inferiortemporal	-2.89
			ctx-rh-rostralmiddlefrontal	-2.23
Imagination	recall	1	Right-Thalamus-Proper	4.85
			ctx-rh-posteriorcingulate	4.79
			Right-Caudate	4.23
			ctx-rh-caudalanteriorcingulate	4.22
			ctx-rh-postcentral	4.05

			ctx-rh-supramarginal	3.87
			ctx-rh-precentral	3.79
			Right-Pallidum	3.66
			ctx-rh-middletemporal	3.52
			ctx-rh-superiorfrontal	3.47
			ctx-rh-superiortemporal	3.34
			Right-Putamen	3.26
			Right-Accumbens-area	3.24
			ctx-rh-transversetemporal	3.24
			ctx-rh-insula	3.23
			ctx-rh-paracentral	3.06
			ctx-lh-entorhinal	2.58
			ctx-rh-caudalmiddlefrontal	2.58
			Right-Amygdala	2.49
			Right-Hippocampus	2.25
		2	ctx-rh-cuneus	-4.62
			ctx-rh-pericalcarine	-4.62
			ctx-rh-precuneus	-4.6
			ctx-rh-isthmuscingulate	-4.41
			ctx-rh-lingual	-4.34
			ctx-rh-fusiform	-4.02
			ctx-rh-lateraloccipital	-3.53
			ctx-rh-inferiorparietal	-2.75

			ctx-rh-inferiortemporal	-2.66
			Right-Hippocampus	-2.29
			ctx-rh-middletemporal	-2.23
	manipulation	1	Right-Thalamus-Proper	4.64
			Right-Caudate	4.21
			Right-Pallidum	3.99
			ctx-rh-parsopercularis	3.39
			ctx-rh-precentral	3.36
			Right-Putamen	3.36
			ctx-rh-caudalmiddlefrontal	3.36
			Right-Accumbens-area	3.34
			Right-Amygdala	3.24
			Right-Hippocampus	3.16
			ctx-rh-postcentral	3.09
			ctx-rh-insula	3.01
			ctx-rh-posteriorcingulate	2.81
			ctx-rh-rostralmiddlefrontal	2.71
			ctx-rh-entorhinal	2.57
			ctx-rh-superiorfrontal	2.55
			ctx-rh-supramarginal	2.46
			ctx-rh-caudalanteriorcingulate	2.11
Third minus first sound	recall	1	ctx-rh-medialorbitofrontal	5.53
			ctx-rh-lateralorbitofrontal	4.8
			ctx-rh-parsorbitalis	4.75

			ctx-rh-rostralanteriorcingulate	4.75
			ctx-rh-parstriangularis	4.36
			ctx-rh-rostralmiddlefrontal	4.34
			Right-Accumbens-area	4.31
			ctx-rh-parsopercularis	4.22
			Right-Caudate	3.96
			ctx-rh-insula	3.58
			ctx-rh-superiortemporal	3.2
			Right-Putamen	3.14
			ctx-rh-superiorfrontal	2.86
			ctx-rh-caudalmiddlefrontal	2.56
			ctx-rh-temporalpole	2.09
		2	ctx-rh-inferiortemporal	-6.15
			ctx-rh-middletemporal	-5.86
			ctx-rh-fusiform	-5.48
			ctx-rh-superiortemporal	-5.24
			ctx-rh-bankssts	-5.22
			ctx-rh-postcentral	-5.01
			ctx-rh-supramarginal	-4.34
			ctx-rh-parahippocampal	-4.17
			Right-Hippocampus	-4.14
			ctx-rh-transversetemporal	-3.89
			ctx-rh-insula	-3.64

			ctx-rh-precentral	-3.51
			Right-Putamen	-3.37
			Right-Thalamus-Proper	-3.29
			Right-Pallidum	-3.25
			ctx-rh-entorhinal	-3.1
			Right-Amygdala	-2.56
			ctx-rh-isthmuscingulate	-2.14
	manipulation	1	ctx-rh-parsorbitalis	4
			ctx-rh-lateralorbitofrontal	3.89
			ctx-rh-parstriangularis	3.78
			ctx-rh-rostralmiddlefrontal	3.74
			Right-Accumbens-area	3.26
			ctx-rh-parsopercularis	3.18
			ctx-rh-rostralanteriorcingulate	3.06
			Right-Caudate	3.02
			ctx-rh-medialorbitofrontal	3.02
			ctx-rh-insula	2.77
			ctx-rh-superiortemporal	2.51
		2	ctx-rh-middletemporal	-5.96
			ctx-rh-bankssts	-5.77
			ctx-rh-superiortemporal	-5.73
			ctx-rh-supramarginal	-5.59
			ctx-rh-inferiortemporal	-5.5

			Right-Thalamus-Proper	-5.26
			Right-Hippocampus	-5.22
			Right-Putamen	-4.91
			Right-Pallidum	-4.86
			ctx-rh-postcentral	-4.72
			ctx-rh-transversetemporal	-4.61
			ctx-rh-parahippocampal	-4.34
			ctx-rh-fusiform	-4.31
			Right-Amygdala	-4.3
			ctx-rh-insula	-4.06
			ctx-rh-precentral	-3.96
			ctx-rh-entorhinal	-3.76
			ctx-rh-inferiorparietal	-3.49
			ctx-rh-posteriorcingulate	-2.8
			ctx-rh-paracentral	-2.79
			Right-Caudate	-2.74
			ctx-rh-isthmuscingulate	-2.26
imagination minus listening	recall	1	Right-Thalamus-Proper	7.56
			Right-Caudate	6.42
			ctx-rh-posteriorcingulate	6.2
			Right-Accumbens-area	5.74
			Right-Pallidum	5.6
			ctx-rh-caudalanteriorcingulate	5.47
			ctx-rh-superiorfrontal	5.42

			Right-Putamen	5.18
			ctx-rh-paracentral	4.91
			Right-Amygdala	4.66
			ctx-rh-postcentral	4.53
			ctx-rh-precentral	4.3
			Right-Hippocampus	4.23
			ctx-rh-middletemporal	4.04
			ctx-rh-caudalmiddlefrontal	4
			ctx-rh-superiortemporal	3.98
			ctx-rh-transversetemporal	3.93
			ctx-rh-insula	3.92
			ctx-rh-supramarginal	3.85
			ctx-rh-medialorbitofrontal	3.74
			ctx-rh-parsopercularis	3.34
			ctx-rh-entorhinal	3.29
			ctx-rh-lateralorbitofrontal	3.1
			ctx-rh-rostralmiddlefrontal	2.57
			ctx-rh-inferiortemporal	2.47
			ctx-rh-parahippocampal	2.46
			ctx-rh-isthmuscingulate	2.45
		2	ctx-rh-lingual	-6.44
			ctx-rh-isthmuscingulate	-6.13
			ctx-rh-precuneus	-5.75

			ctx-rh-pericalcarine	-5.25
			ctx-rh-cuneus	-5.2
			ctx-rh-lateraloccipital	-4.24
			ctx-rh-fusiform	-4.14
			ctx-rh-inferiortemporal	-3.88
			ctx-rh-middletemporal	-3.61
			ctx-rh-inferiorparietal	-3.61
			ctx-rh-parahippocampal	-3.44
			Right-Hippocampus	-3.24
			ctx-rh-bankssts	-2.7
			ctx-rh-superiorparietal	-2.46
	manipulation	1	Right-Thalamus-Proper	6.25
			Right-Caudate	4.95
			ctx-rh-posteriorcingulate	4.66
			ctx-rh-caudalmiddlefrontal	4.58
			Right-Pallidum	4.54
			ctx-rh-parsopercularis	4.53
			Right-Putamen	4.44
			ctx-rh-superiorfrontal	4.38
			ctx-rh-caudalanteriorcingulate	4.35
			Right-Accumbens-area	4.3
			ctx-rh-precentral	4.11
			ctx-rh-insula	3.71

			Right-Amygdala	3.29
			ctx-rh-paracentral	3.27
			ctx-rh-rostralmiddlefrontal	3.21
			ctx-rh-postcentral	3.07
			Right-Hippocampus	3.02
			ctx-rh-medialorbitofrontal	2.79
			ctx-rh-lateralorbitofrontal	2.52
			ctx-rh-parstriangularis	2.43

Table E. Peak t-value for each region in the **left** hemisphere that overlapped with significant clusters in each of the tested periods and conditions. The Desikan-Killiany parcellation was used to obtain anatomic labels.

Period / comparison	Condition	Cluster No	label	t value
First sound	recall	1	ctx-lh-precuneus	4.46
			ctx-lh-bankssts	4.34
			ctx-lh-middletemporal	4.28
			ctx-lh-inferiortemporal	4.17
			ctx-lh-fusiform	4.15
			ctx-lh-cuneus	4
			ctx-lh-inferiorparietal	3.88
			ctx-lh-superiorparietal	3.87
			ctx-lh-supramarginal	3.57
			ctx-lh-superiortemporal	3.38
			ctx-lh-parahippocampal	3.31

			ctx-lh-lingual	3
			ctx-lh-isthmuscingulate	2.92
			Left-Hippocampus	2.87
			ctx-lh-pericalcarine	2.75
			ctx-lh-lateraloccipital	2.73
		2	Left-Accumbens-area	-7.4
			Left-Caudate	-7.1
			Left-Thalamus-Proper	-6.72
			ctx-lh-medialorbitofrontal	-6.36
			Left-Pallidum	-6.23
			Left-Putamen	-6.15
			ctx-lh-rostralanteriorcingulate	-5.97
			ctx-lh-lateralorbitofrontal	-5.45
			ctx-lh-insula	-5.18
			ctx-lh-parsopercularis	-4.99
			ctx-lh-parstriangularis	-4.95
			ctx-lh-caudalmiddlefrontal	-4.1
			ctx-lh-rostralmiddlefrontal	-3.99
			Left-Amygdala	-3.83
			ctx-lh-posteriorcingulate	-3.67
			ctx-lh-precentral	-3.39
			ctx-lh-parsorbitalis	-3
			Left-Hippocampus	-3

			ctx-lh-superiortemporal	-2.95
			ctx-lh-inferiortemporal	-2.91
			ctx-lh-entorhinal	-2.89
			ctx-lh-temporalpole	-2.85
			ctx-lh-caudalanteriorcingulate	-2.4
			ctx-lh-fusiform	-2.32
			ctx-lh-isthmuscingulate	-2.15
			ctx-lh-precuneus	-2.06
	manipulation	1	ctx-lh-middletemporal	5.14
			ctx-lh-bankssts	5.04
			ctx-lh-inferiorparietal	4.86
			ctx-lh-inferiortemporal	4.71
			ctx-lh-lateraloccipital	4.64
			ctx-lh-fusiform	4.62
			ctx-lh-lingual	3.76
			ctx-lh-superiortemporal	3.68
			ctx-lh-supramarginal	3.62
			ctx-lh-parahippocampal	3.42
			Left-Hippocampus	2.93
			ctx-lh-superiorparietal	2.8
			ctx-lh-isthmuscingulate	2.54
			ctx-lh-precuneus	2.49
			ctx-lh-cuneus	2.29

		3	Left-Caudate	-5.72
			Left-Thalamus-Proper	-5.56
			Left-Putamen	-5.45
			Left-Accumbens-area	-5.34
			Left-Pallidum	-5.17
			ctx-lh-insula	-4.95
			ctx-lh-parsopercularis	-4.76
			ctx-lh-medialorbitofrontal	-4.63
			ctx-lh-parstriangularis	-4.49
			ctx-lh-rostralanteriorcingulate	-4.36
			ctx-lh-caudalmiddlefrontal	-3.84
			ctx-lh-lateralorbitofrontal	-3.82
			ctx-lh-precentral	-3.78
			ctx-lh-rostralmiddlefrontal	-3.38
			Left-Amygdala	-3.35
			ctx-lh-posteriorcingulate	-3.07
			ctx-lh-postcentral	-3.06
			ctx-lh-superiortemporal	-2.95
			Left-Hippocampus	-2.77
			ctx-lh-caudalanteriorcingulate	-2.35
			ctx-lh-entorhinal	-2.3
			ctx-lh-temporalpole	-2.28
			ctx-lh-supramarginal	-2.11
Second sound	recall	1	ctx-lh-fusiform	4.73

			ctx-lh-lingual	4.26
			ctx-lh-parahippocampal	4.21
			ctx-lh-isthmuscingulate	4.06
			ctx-lh-precuneus	3.76
			Left-Hippocampus	3.75
			ctx-lh-inferiortemporal	3.31
			ctx-lh-pericalcarine	3.21
			ctx-lh-cuneus	3.15
			ctx-lh-superiorparietal	3.15
			ctx-lh-middletemporal	2.93
			ctx-lh-bankssts	2.69
			ctx-lh-superiortemporal	2.65
			ctx-lh-supramarginal	2.55
		2	Left-Thalamus-Proper	-4.58
			ctx-lh-posteriorcingulate	-4.03
			Left-Caudate	-3.54
			Left-Accumbens-area	-3.28
			ctx-lh-caudalanteriorcingulate	-2.81
			Left-Pallidum	-2.68
			ctx-lh-isthmuscingulate	-2.54
			ctx-lh-medialorbitofrontal	-2.33
			Left-Putamen	-2.21
			ctx-lh-rostralanteriorcingulate	-2.18

	manipulation	1	ctx-lh-precentral	-4.56
			ctx-lh-caudalmiddlefrontal	-3.77
			ctx-lh-postcentral	-3.58
			ctx-lh-caudalanteriorcingulate	-3.51
			Left-Thalamus-Proper	-3.43
			ctx-lh-posteriorcingulate	-3.36
			ctx-lh-superiorfrontal	-3.34
			Left-Pallidum	-2.94
			ctx-lh-inferiortemporal	-2.82
			ctx-lh-paracentral	-2.71
			Left-Caudate	-2.63
			Left-Amygdala	-2.61
			Left-Putamen	-2.59
			Left-Hippocampus	-2.52
			ctx-lh-superiortemporal	-2.37
			ctx-lh-middletemporal	-2.1
			ctx-lh-fusiform	-2.09
Third sound	recall	1	ctx-lh-lingual	3.08
			ctx-lh-precuneus	2.76
			ctx-lh-cuneus	2.71
			ctx-lh-lateraloccipital	2.69
			ctx-lh-pericalcarine	2.64
			ctx-lh-isthmuscingulate	2.5
		2	Left-Thalamus-Proper	-5

			ctx-lh-superiortemporal	-4.83
			ctx-lh-transversetemporal	-4.7
			ctx-lh-postcentral	-4.6
			ctx-lh-supramarginal	-4.4
			ctx-lh-bankssts	-4.35
			ctx-lh-middletemporal	-4.21
			ctx-lh-insula	-3.54
			ctx-lh-inferiortemporal	-3.51
			ctx-lh-precentral	-3.35
			Left-Caudate	-3.12
			Left-Putamen	-2.71
			ctx-lh-inferiorparietal	-2.7
			ctx-lh-fusiform	-2.59
			ctx-lh-posteriorcingulate	-2.58
			Left-Hippocampus	-2.41
			Left-Pallidum	-2.21
			ctx-lh-parahippocampal	-2.15
	manipulation	1	ctx-lh-superiortemporal	-4.66
			ctx-lh-supramarginal	-4.54
			ctx-lh-bankssts	-4.23
			Left-Thalamus-Proper	-4.02
			ctx-lh-middletemporal	-3.98
			ctx-lh-postcentral	-3.96

			ctx-lh-precentral	-3.83
			ctx-lh-transversetemporal	-3.33
			Left-Hippocampus	-2.89
			Left-Amygdala	-2.85
			ctx-lh-inferiortemporal	-2.79
			ctx-lh-parahippocampal	-2.66
			ctx-lh-insula	-2.61
			ctx-lh-entorhinal	-2.58
			Left-Caudate	-2.51
			ctx-lh-fusiform	-2.43
			Left-Pallidum	-2.42
			Left-Putamen	-2.27
Imagination	recall	1	Left-Thalamus-Proper	4.95
			ctx-lh-posteriorcingulate	4.93
			Left-Pallidum	4.14
			ctx-lh-caudalanteriorcingulate	4.05
			Left-Caudate	4
			Left-Putamen	3.51
			Left-Accumbens-area	3.41
			ctx-lh-precentral	3.15
			Left-Amygdala	3.07
			Left-Hippocampus	3.04
			ctx-lh-superiorfrontal	2.91
			ctx-lh-insula	2.68

			ctx-lh-caudalmiddlefrontal	2.63
			ctx-lh-entorhinal	2.58
			ctx-lh-rostralmiddlefrontal	2.51
			ctx-lh-paracentral	2.44
			ctx-lh-superiortemporal	2.4
			ctx-lh-postcentral	2.14
		2	ctx-lh-precuneus	-4.37
			ctx-lh-cuneus	-4.34
			ctx-lh-isthmuscingulate	-4.32
			ctx-lh-pericalcarine	-3.95
			ctx-lh-lingual	-3.49
			ctx-lh-middletemporal	-3.37
			ctx-lh-lateraloccipital	-3.37
			ctx-lh-inferiorparietal	-3.37
			ctx-lh-bankssts	-3.3
			ctx-lh-superiorparietal	-3.09
			ctx-lh-superiortemporal	-2.95
			ctx-lh-supramarginal	-2.93
			Left-Hippocampus	-2.78
			ctx-lh-inferiortemporal	-2.74
			ctx-lh-parahippocampal	-2.32
			ctx-lh-fusiform	-2.22
	manipulation	1	Left-Thalamus-Proper	3.91

			ctx-lh-entorhinal	2.37
			ctx-lh-parahippocampal	2.37
			Left-Hippocampus	2.34
			Left-Amygdala	2.24
	difference	1	ctx-lh-parsopercularis	-3.34
			ctx-lh-caudalmiddlefrontal	-3.05
			ctx-lh-precentral	-2.88
			ctx-lh-insula	-2.8
			ctx-lh-rostralmiddlefrontal	-2.78
			ctx-lh-parstriangularis	-2.76
			ctx-lh-lateralorbitofrontal	-2.76
			Left-Thalamus-Proper	-2.71
			Left-Putamen	-2.66
			ctx-lh-superiorfrontal	-2.65
			Left-Caudate	-2.58
			ctx-lh-parsorbitalis	-2.49
			Left-Pallidum	-2.48
			ctx-lh-posteriorcingulate	-2.1
			ctx-lh-superiortemporal	-2.05
Third minus first sound	recall	1	ctx-lh-medialorbitofrontal	5.11
			ctx-lh-rostralanteriorcingulate	4.82
			Left-Accumbens-area	4.6
			ctx-lh-lateralorbitofrontal	4.52
			Left-Putamen	4.12

			Left-Caudate	4.09
			ctx-lh-insula	3.69
			Left-Pallidum	3.44
			ctx-lh-parstriangularis	3.23
			ctx-lh-parsorbitalis	3.16
			ctx-lh-parsopercularis	3.08
			ctx-lh-superiortemporal	3.07
			ctx-lh-rostralmiddlefrontal	2.96
			ctx-lh-superiorfrontal	2.89
			ctx-lh-temporalpole	2.72
			ctx-lh-caudalmiddlefrontal	2.4
			Left-Thalamus-Proper	2.2
		2	Left-Thalamus-Proper	-2.31
		3	ctx-lh-middletemporal	-4.78
			ctx-lh-bankssts	-4.65
			ctx-lh-superiortemporal	-4.53
			ctx-lh-inferiorparietal	-4.41
			ctx-lh-inferiortemporal	-4.28
			ctx-lh-supramarginal	-3.99
			ctx-lh-fusiform	-3.95
			ctx-lh-postcentral	-3.44
			ctx-lh-transversetemporal	-3.32
			Left-Hippocampus	-3.01

			ctx-lh-parahippocampal	-2.97
			ctx-lh-insula	-2.55
	manipulation	1	Left-Caudate	3.92
			ctx-lh-insula	3.88
			ctx-lh-parstriangularis	3.78
			Left-Putamen	3.57
			ctx-lh-parsopercularis	3.53
			ctx-lh-medialorbitofrontal	3.42
			ctx-lh-lateralorbitofrontal	3.31
			ctx-lh-rostralanteriorcingulate	3.3
			Left-Accumbens-area	3.24
			ctx-lh-superiorfrontal	2.92
			ctx-lh-precentral	2.88
			Left-Pallidum	2.82
			ctx-lh-parsorbitalis	2.72
			ctx-lh-rostralmiddlefrontal	2.62
			ctx-lh-postcentral	2.54
			ctx-lh-superiortemporal	2.47
			ctx-lh-caudalmiddlefrontal	2.37
		2	ctx-lh-middletemporal	-5.19
			ctx-lh-bankssts	-5.11
			ctx-lh-inferiortemporal	-4.65
			ctx-lh-superiortemporal	-4.45

			ctx-lh-inferiorparietal	-3.99
			ctx-lh-supramarginal	-3.77
			ctx-lh-fusiform	-3.5
			ctx-lh-parahippocampal	-3.24
			Left-Hippocampus	-3.19
			ctx-lh-transversetemporal	-2.87
			ctx-lh-lingual	-2.82
			ctx-lh-isthmuscingulate	-2.23
imagination minus listening	recall	1	Left-Thalamus-Proper	8.23
			Left-Caudate	6.94
			Left-Pallidum	6.24
			Left-Accumbens-area	6.22
			ctx-lh-posteriorcingulate	6.18
			Left-Putamen	5.14
			ctx-lh-caudalanteriorcingulate	4.64
			Left-Amygdala	4.44
			Left-Hippocampus	4.17
			ctx-lh-precentral	3.93
			ctx-lh-medialorbitofrontal	3.52
			ctx-lh-isthmuscingulate	3.38
			ctx-lh-inferiortemporal	3.36
			ctx-lh-postcentral	3.31
			ctx-lh-entorhinal	3.12
			ctx-lh-caudalmiddlefrontal	2.91

			ctx-lh-insula	2.89
			ctx-lh-superiortemporal	2.86
			ctx-lh-rostralanteriorcingulate	2.81
			ctx-lh-transversetemporal	2.79
			ctx-lh-fusiform	2.72
			ctx-lh-lateralorbitofrontal	2.65
			ctx-lh-rostralmiddlefrontal	2.65
			ctx-lh-superiorfrontal	2.6
			ctx-lh-parsopercularis	2.22
			ctx-lh-parstriangularis	2.2
			ctx-lh-supramarginal	2.09
		2	ctx-lh-precuneus	-6.5
			ctx-lh-cuneus	-5.67
			ctx-lh-superiorparietal	-5.3
			ctx-lh-isthmuscingulate	-5.22
			ctx-lh-fusiform	-5.01
			ctx-lh-pericalcarine	-4.99
			ctx-lh-middletemporal	-4.37
			ctx-lh-bankssts	-4.32
			ctx-lh-inferiorparietal	-4.29
			ctx-lh-lingual	-4.19
			ctx-lh-inferiortemporal	-4.17
			ctx-lh-supramarginal	-3.8

			ctx-lh-parahippocampal	-3.7
			ctx-lh-lateraloccipital	-3.6
			ctx-lh-superiortemporal	-3.09
			Left-Hippocampus	-2.6
	manipulation	1	Left-Thalamus-Proper	6.52
			Left-Pallidum	4.53
			Left-Accumbens-area	4.5
			Left-Caudate	4.14
			Left-Amygdala	3.71
			Left-Putamen	3.68
			Left-Hippocampus	3.41
			ctx-lh-posteriorcingulate	3.07
			ctx-lh-superiorfrontal	2.93
			ctx-lh-entorhinal	2.89
			ctx-lh-paracentral	2.82
			ctx-lh-middletemporal	2.74
			ctx-lh-parahippocampal	2.67
			ctx-lh-fusiform	2.66
			ctx-lh-insula	2.63
			ctx-lh-superiortemporal	2.61
			ctx-lh-transversetemporal	2.58
			ctx-lh-medialorbitofrontal	2.57
			ctx-lh-caudalanteriorcingulate	2.39

References

1. R. S. Desikan, *et al.*, An automated labeling system for subdividing the human cerebral cortex on MRI scans into gyral based regions of interest. *Neuroimage* **31**, 968–980 (2006).
2. D. Bates, M. Mächler, B. Bolker, S. Walker, Fitting Linear Mixed-Effects Models Using lme4. *Journal of Statistical Software* **67** (2015).
3. R Core Team, *R: A Language and Environment for Statistical Computing* (R Foundation for Statistical Computing, 2019).
4. A. Kuznetsova, P. B. Brockhoff, R. H. B. Christensen, lmerTest Package: Tests in Linear Mixed Effects Models. *J. Stat. Soft.* **82** (2017).
