# Supplementary material for: Decoding reveals the neural representation of perceived and imagined musical sounds
Source: PLoS Biol. 2024 Oct 21;22(10):e3002858. doi: 10.1371/journal.pbio.3002858 (PMC11527242; doi:10.1371/journal.pbio.3002858)
Supplement: Attachment — Submitted filename: Review.pdf [file pbio.3002858.s008.pdf]

Dear Dr. Schnell,

Dear Authors,

I thoroughly enjoyed reviewing "***Decoding reveals the neural representation of perceived and imagined musical thoughts***" submitted for publication in "*PLOS Biology*" (review request received: 27 June 2024, review accepted: 01 July 2024, review completed: 08 July 2024).

This research opens a new door in understanding the perceived and imagined melodies from MEG data and is novel in its contribution to the current field researching imagination, music, and the brain. Particularly, MVPA analysis adds a new flavour to this investigation, which contributes to other neuroimaging techniques being employed, specifically studying the auditory cortex.

In general, the work is very well written, is effective in conveying the information directly to the reader and fits within the scope of "*PLOS Biology*", and I recommend this manuscript for publication as it presents a valuable impact to the field. It is already in great shape; however, I have some comments that I recommend the authors should address in revision. I intend these comments as constructive feedback to support and enhance your efforts.

Comments

Abstract

Comment: "With little effort you could sing it or play it vividly in your mind." I find this sentence too generalised. While many people can do this, this ability may not come naturally to everyone. I guess with *could*, you tried to convey this, however, I believe rephrasing it may enhance clarity.

Introduction

Comment: The "Happy birthday" example is very commonly used. It is effective, but reference(s) are needed here, e.g.,

Cotter, K. N., Christensen, A. P., & Silvia, P. J. (2019). Understanding inner music: A dimensional approach to musical imagery. *Psychology of Aesthetics, Creativity, and the Arts*, *13*(4), 489.

Küssner, M. B., Taruffi, L., & Floridou, G. A. (Eds.). (2023). *Music and Mental Imagery*. London and New York: Routledge.

Halpern, A. R. (2012). Dynamic aspects of musical imagery. *Annals of the New York Academy of Sciences*, *1252*(1), 200-205.

Poliakoff, E., Bek, J., Phillips, M., Young, W. R., & Rose, D. C. (2023). Vividness and Use of Imagery Related to Music and Movement in People with Parkinson's: A Mixed-methods Survey Study. *Music & Science*, *6*, 20592043231197919.

Di Liberto, G. M., Marion, G., & Shamma, S. A. (2021). Accurate decoding of imagined and heard melodies. *Frontiers in Neuroscience*, *15*, 673401.

Comment: p. 13, fMRI and EEG to be spelled out the first time.

Comment: Introduction lacks literature that is current and related to this study, such as auditory imagery with or without MEG. For instance, very relevant works include:

Herff, S. A., Herff, C., Milne, A. J., Johnson, G. D., Shih, J. J., & Krusienski, D. J. (2020). Prefrontal High Gamma in ECoG tags periodicity of musical rhythms in perception and imagination. *Eneuro*, *7*(4).

Dash, D., Ferrari, P., & Wang, J. (2020). Decoding imagined and spoken phrases from non-invasive neural (MEG) signals. *Frontiers in neuroscience*, *14*, 290.

Cheng, T. H. Z., Creel, S. C., & Iversen, J. R. (2022). How do you feel the rhythm: Dynamic motor-auditory interactions are involved in the imagination of hierarchical timing. *Journal of Neuroscience*, *42*(3), 500-512.

Schaefer, R. S. (2014). Images of time: Temporal aspects of auditory and movement imagination. *Frontiers in Psychology*, *5*, 877.

Dijkstra, N., Kok, P., & Fleming, S. M. (2022). Perceptual reality monitoring: Neural mechanisms dissociating imagination from reality. *Neuroscience & Biobehavioral Reviews*, *135*, 104557.

Dijkstra, N., & Fleming, S. M. (2023). Subjective signal strength distinguishes reality from imagination. *Nature Communications*, *14*(1), 1627.

Intro can also benefit from more primary music-related imagery work that is associated with your work, as there are many imagery modalities – auditory, vision -, and this body of literature below, for instance, is currently investigating specific/overall modalities. It is worth pointing out here what imagery modalities you exactly are investigating. If that is not modality-specific, that is also okay, but I cannot be exactly sure whether it is auditory or other modalities as well that you are investigating here from the text, or the definition of the “imagination” is not too clear.

Küssner, M. B., & Taruffi, L. (2022). Modalities and causal routes in music-induced mental imagery. *Trends in Cognitive Sciences*.

Taruffi, L., Ayyildiz, C., & Herff, S. A. (2023). Thematic Contents of Mental Imagery are Shaped by Concurrent Task-Irrelevant Music. *Imagination, Cognition and Personality*, *43*(2), 169-192.

Herff, S. A., Cecchetti, G., Taruffi, L., & Déguernel, K. (2021). Music influences vividness and content of imagined journeys in a directed visual imagery task. *Scientific Reports*, *11*(1), 15990.

Herff, S. A., McConnell, S., Ji, J. L., & Prince, J. B. (2022). Eye Closure Interacts with Music to Influence Vividness and Content of Directed Imagery. *Music & Science*, *5*, 20592043221142711.

Margulis, E. H., & Jakubowski, K. (2024). Music, Memory, and Imagination. *Current Directions in Psychological Science*, *33*(2), 108-113.

Margulis, E. H., & McAuley, J. D. (2022). Mechanisms and individual differences in music-evoked imaginings. *Trends in Cognitive Sciences*.

Comment: "However, no study has elucidated **1)** the nature of sound sequence representations in auditory and association areas, **2)** how these representations evolve over time and **3)** how they change when mentally manipulated." - "***no study***" is simply untrue and very argumentative within this sentence as there are many studies looking at these aims (such as above and some of the work you've already cited). Although your study is novel in its aims noted elsewhere, please change the wording here or change the sentence in a way that no study studied what you exactly are looking at here.

Results

Comment: "Participants (N = 71, 44 female, age = 28.77 +/- 8.43 SD) performed with high accuracy (**Fig. 1c**) and were better (*OR* = 1.85, CI = [1.25 - 2.72], *p* = .002) in the recall (96.7%, CI = [95.6 - 97.6]) than the manipulation (94.1%, CI = [91.9 - 95.8]) block. This confirms the presence of melody specific information during the imagination period." – This finding can also be interpreted in other ways, such as differences in task difficulty or participant engagement between the recall and manipulation blocks that might be crucial to understand the differences of the recall and manipulation blocks. Feel free to ignore this, but maybe, it is worth pointing out in the discussion.

Comment: "This is indicative of a flip in neural representations such that models trained in one condition were consistently "wrong" when tested on the other condition." – Here, I feel "wrong" is the wrong word to use here. Possibly, "inaccurate" might be a better word as it is more precise and scientifically appropriate in the context of neural representations and model testing.

Comment: Sound and tones are interchangeably used. It is worth keeping these terms consistent.

Discussion

Comment: 2nd and 3rd paragraph of the discussion could be combined as the 2nd paragraph feels incomplete, and they seem to be very short paragraphs. There are missing references too, and it is unclear whether the last sentences are speculation or not to the general reader, so references would go a long way here.

Comment: **"**Temporal order is an abstract feature that might generalize less across listening and imagination than sensory features such as pitch, which are typically the target of decoding."- missing reference here. E.g.,

Sankaran, N., Thompson, W. F., Carlile, S., & Carlson, T. A. (2018). Decoding the dynamic representation of musical pitch from human brain activity. *Scientific reports*, *8*(1), 839.

Comment: I'd also include references to the omission studies examples as well as for the fMRI ones.

Comment: "…which might introduce between-trial and between-subject variations that blur sound-specific representations.". – I think it could be important to highlight how this could be addressed, such as including more trials to enhance the reliability of the data, or considering using mixed-effects models to account for both fixed (conditions) and random (participant variability). I guess running mixed-effects models could address this limitation most straightforwardly within this paper, but that is up to the authors' choice, as simply addressing the limitation and how to address it in the future is also okay.

Comment: "previous neuroimaging activation studies as important for auditory imagery." – Is this only relevant for auditory imagery or other modalities too? Or is there something special related to auditory imagery here?

Comment: "Furthermore, we observed melody specific representations in the basal nuclei, a set of areas involved in both cognitive and motor control that have not been identified in previous fMRI research" – Here, I find "previous fMRI research" very specific. It might be worth clarifying which fMRI research, such as including "… fMRI research on melody processing." And I might be missing something here, but why only fMRI research? How about EEG, etc., or is this only an example?

Comment: "Finally, our results demonstrate the feasibility…". – I would change "Finally" to "Overall" as finally read like it is the final point you want to make. This paragraph could take advantage of adding a sentence regarding the purpose of the study and how this demonstrated in your results.

Method

Comment: For stimuli, no need for "timbre" for piano, as this can be confusing to the general reader if the definition of timbre is not being used before (as it is a complex subject and definition is ill-defined and in debate (Siedenburg et al., 2019), just leaving "piano" would be sufficient.

Comment: The stimuli section is too brief. It lacks information on whether the stimuli are recorded (if it was, how?), or taken from elsewhere, or produced/ artificially made? Did the piano sound natural or not (e.g., artificially made)? Currently, one cannot replicate re-producing the stimuli.

Comment: melody1 and melody2 are written as melody 1 and melody 2 at different points of the Method section.

Comment: Regarding the task, how long did the experiment take?

Comment: "using the inverse solutions described above (**Fig. 1f**)"- in Figure 1f, instead of above.

Comment: For completeness, I liked the addition of relationship with behaviour. Is there a missing citation here: "(R, lme4; , 52, 53)? However, it might also be worth adding this section to the supplementary materials instead of here as this does not significantly add to the main points. Words could be used more to improve the Introduction and Discussion section.

***Comma Comments*:**

- Oxford comma inconsistency
- Missing commas (e.g., "Interestingly, after the second sound, melodies were discriminated in association, sensorimotor and subcortical structures ($p < .005$), but not in auditory areas (**Fig. 3b**).

Thank you a lot for your fascinating contribution, and I look forward to seeing a revised and published version of this study.
