# Supplementary material for: Decoding reveals the neural representation of perceived and imagined musical sounds
Source: PLoS Biol. 2024 Oct 21;22(10):e3002858. doi: 10.1371/journal.pbio.3002858 (PMC11527242; doi:10.1371/journal.pbio.3002858)
Supplement: Attachment — Submitted filename: R2R_round1.pdf [file pbio.3002858.s009.pdf]

Response to reviewers

We thank the reviewers for taking the time to read and evaluate our manuscript in such depth. We appreciate their comments, which helped us improve the manuscript, and are very grateful for the positive stance towards our work. Below we reply to each of the points raised. We have tagged each comment with reviewer and comment number (e.g., R2.2) and provide a response immediately after.

Reviewer #1: This study aims to characterize the neural representations of imagined melodies. Participants are tasked to recall and manipulate simple three-note melodies while their MEG is measured. Using a combination of multivariate pattern analysis and source reconstruction, the authors find significant representations of imagined (both recalled and manipulated) melodies throughout several association areas. Furthermore, interesting sign flips are found between perception and imagination, providing important avenues for future research.

The paper is well-written and uses advanced analysis in a comprehensive way to study an important question about the neural mechanisms of auditory imagination. However, I do have several methodological queries that need to be addressed.

Major comments:

R1.1 Due to the experimental design, in the recall block, imagine A is always preceded by listen A and imagine B is always preceded by listen B. Therefore, is it possible that (part of) the results are due to temporal autocorrelations in the signal between listening and imagining? I appreciate that this cannot explain the between-condition negative decoding, but could it explain within-condition decoding as well as part of the source analysis results?

Response: It is unlikely that successful within-condition decoding during the imagination period is due to temporal autocorrelations between listening and imagination for the following reasons:

1- The neural response evoked by a sound is short-lived with typical sensory/perceptual components fading before 1s (1). In contrast, here above-chance decoding was sustained at a similar level during the whole imagination period, up to three seconds after the onset of the third sound in the melody.
2- The decoding models were trained for each time point in the trial **separately** and **independently** so that no training set included samples from the listening and imagination periods in the same model.
3- Following the temporal generalization technique, training in the listening period and testing in the imagination period (or vice versa) yielded no above chance accuracy, indicating that listening and imagination had different neural representations.
4- Training in one condition (e.g., recall) and testing on the other condition (e.g., manipulation) yielded similar accuracy for the listening period, but statistically different accuracy in the imagination period, further suggesting that representations were different between the two periods.

Regarding source reconstruction, we have included an additional analysis in the supplement (Fig S8) where we use the data from the listening and imagination periods **separately** to estimate the inverse solution, which yielded similar results to the original analyses.

R1.2 The behavioural results of a ceiling effect are taken to 'confirm the presence of melody specific information during the imagination period'. However, unless I missed something, it could just as well reflect accurate memory of the 'listen' period, right? Especially as the accuracy is higher during the

recall compared to the manipulation blocks. Relatedly, without a control condition in which participants were not explicitly asked to 'imagine as vividly as possible' it is hard to know which of the results reflect conscious imagery versus (potentially unconscious) working memory. This is important because recent studies with people who are unable to imagine (aphantasia) suggest that the two might rely on different neural mechanisms (see e.g. Keogh et al., 2021 Cortex).

Response: We agree with the reviewer that we cannot be entirely sure that participants vividly imagined the melodies in all trials of the task, and that other (possibly unconscious) retention mechanisms might have been used. The inclusion of a non-imagery condition would have been ideal, but due to logistic and resource constraints we prioritized our main question of interest, namely the difference in neural representations between recall and manipulation. However, we also note that between-condition decoding accuracies in the imagination period provide evidence that participants actively engaged in the mental recall and manipulation of sound sequences. Furthermore, after the task, we asked participants to rate the vividness of their imagery during the experiment, with most participants reporting mild-to-strong vividness. We have modified the relevant passage in section 2.1. to include this information:

> *“After the experiment, participants rated task-related imagery vividness on a 7-point Likert scale (from -3 to 3), with 72% of them rating 0 or above, which ranges from mild to strong vividness (Table 1). The good task performance and the vividness ratings suggest the presence of melody-specific information during imagination.”*

We added a summary of the ratings in Table 1:

Table 1. *Vividness ratings at the end of the task across participants.*

Rating	-3	-2	-1	0	1	2	3
**Number of participants**	1	4	8	10	25	12	4
**Percentage**	1%	6%	11%	14%	35%	17%	6%

We have also included language in the discussion making this limitation more explicit:

> *“Thus, while it is possible that our participants used unconscious maintenance strategies without imagery, the explicit task instruction, the good task performance, the vividness ratings, and the between-condition decoding results suggest that they engaged in active mental recall and manipulation. Future experiments where imagery is not required are needed to further elucidate the nature of maintenance strategies and the relationship of imagery with working memory.”*

R1.3 It was unclear to me how exactly the decoding weights/coefficients were transformed to 'activity' patterns for source localisation. In most cases, decoding weights do not only reflect which sensors/sources contribute stimulus information, but large weights can also be obtained even if the associated sensors/sources do not contain stimulus info, e.g. because they are used for noise suppression by the decoding algorithm. More explanation is necessary here to ensure that the authors have transformed the decoding coefficients accurately. For an extensive discussion on this topic, see Haufe et al., 2014 NeuroImage.

Response: To interpret the weights of decoding models, we transformed them using the same method proposed by Haufe et al., 2014 that the reviewer cites. We have made this more explicit in the methods section:

> *"We transformed decoding coefficients (**W**) into interpretable patterns of activation (**A**) for each participant using the method detailed in (35) and defined by the equation:*
>
> $$\boldsymbol{A} = \Sigma_x \boldsymbol{W} \Sigma_{\hat{y}}^{-1}$$
>
> *Where $\Sigma_{\hat{y}}$ is the covariance of* model predictions and Σ_x is the covariance of neural signals."

R1.4 What does 'permutation-corrected' mean in reference to testing for significance of decoding? In general, decoding accuracy is known not to meet all the assumptions of parametric statistical testing and therefore permutation-based methods are more suitable, for more info, see Stelzer et al., 2013 NeuroImage.

Response: We have used the same permutation-based, non-parametric methods that the reviewer suggests. We rephrased the paragraph in the methods where we describe the approach to make it clearer:

> *"At the group level, we used non-parametric cluster-based permutations (67) to evaluate whether accuracies in the time-generalization matrices were significantly above or below chance. Here, chance level corresponds to 0.5 accuracy, as we classified binary melody identity from brain data. We used a two-sided cluster-defining threshold of $p = 0.05$ based on one-sample t-tests ($p = 0.025$ one-sided, $t > 1.99$), and max sum as the cluster statistic. The cluster-level significance threshold was set at $p = .05$. The number of permutations was 5000. The same statistical approach was used to evaluate whether within-condition accuracy was different from between-condition accuracy."*

Minor comments:

R1.5 Please mention in the legend that the dashed lines in Fig. 1F reflect the onset of the different tones.

Response: Done

R1.6 I applaud the large sample size but it also made me wonder whether this was initially set-up as a group-study, given that a portion of the participants had zero musical training whereas others had a median of 11 years musical training. Is that the case? And if so, please report this. Out of interest, were there any differences in decoding depending on musical training?

Response: We did not intend this to be a group study and musicianship was not a factor in recruitment. The number of musicians and non-musicians was not predefined and follows natural variation in the population. In an additional supplementary analysis, we find no significant associations between musicianship and decoding performance (see Table S1). We have added some language in this regard in the methods section:

> *"...Musical expertise was not a factor in recruitment for this experiment. Participants gave their explicit informed consent and received a small monetary compensation."*

Furthermore, we took the opportunity to investigate the relationship of neural discrimination with vividness and behavioral accuracy as well. Here we find hints of a possible association between

neural discrimination and behavioral accuracy for the manipulation block. However, we don't want to over-interpret this result due to the weak effect and the ceiling in task performance. We now refer to these analyses in the first paragraph of section 2.1. and report them in the supplement:

"Exploratory analysis: Relationship of neural decoding accuracy with behavioral measures

*For completeness, in exploratory analyses, we tested whether neural decoding accuracy was related to behavioral accuracy, musical training or vividness ratings. To this purpose, we took the mean of neural decoding accuracy scores in the generalization matrices (**Fig. 2**) for the listening (0s - 2s) and imagination (2s - 4s) periods separately (i.e., the left-bottom and top-right quadrants). Since below-chance and above-chance accuracy values were present, we derived an unsigned neural discrimination measure (**ND**) by subtracting 0.5 (i.e., the chance level) and computing the absolute value before taking the mean of the corresponding period. ND represents the information in the neural signal that drives above or below-chance decoding. In the manipulation condition, ND was significantly correlated with behavioral accuracy for the listening period ($r = .24$, $p = .045$) and marginally for the imagination period ($r = .22$, $p = .052$), before multiple comparisons corrections (**Table S1**). No other significant associations were found.*

Table S1. *Pearson's correlation coefficients (**r**), and corresponding p-values with (**p_{FDR}**) and without (**p**) false-discovery-rate correction, between neural discrimination (**ND**) and three variables of interest: Behavioral accuracy, vividness ratings and music training (GMSI scores)."*

Condition / Period	Behavioral Accuracy			Vividness Ratings			Music Training (GMSI)		
	r	p	p_{FDR}	r	p	p_{FDR}	r	p	p_{FDR}
Recall / listening	-.07	.553	.885	.08	.523	.885	0	.989	.989
Recall / imagination	-.15	.224	.674	.16	.198	.673	.06	.639	.885
Manipulation / listening	**.24**	**.045**	.315	-.01	.924	.989	.1	.425	.885
Manipulation / imagination	.23	.052	.315	-.04	.763	.916	.05	.664	.885

R1.7 How many blocks were there in total?

Response: There were two blocks, one for recall and one for manipulation. We made this explicit in the methods section:

"There were two conditions, encompassing the two different blocks in the experiment."

R1.8 It took me a while to understand that the manipulation block trials were labelled based on the listening period, not on the imagination period, and that below-chance accuracies were therefore expected. It would help the reader if this was made more explicit and clearer in the text.

Response: We have added some language in section 2.2 to make this clearer:

"We used between-condition testing to decode the identity of the perceived melody at all timepoints in the trial and detect manipulation-related changes in neural representations. Thus, if during manipulation participants inhibited the representation of the perceived melody and reinstated the representation of its backward version, between-condition tests should

systematically predict the opposite of the perceived melody, resulting in below-chance accuracy in the imagination period. Indeed, we found below-chance accuracy both when training on recall and testing on manipulation ($p \leq .048$) and when training on manipulation and testing on recall ($p \leq .018$; Fig. 2b; Table S1). In both cases, accuracies were lower for between-condition than within-condition testing ($p \leq .035$; Fig. 2c). This indicates a flip in neural representations such that models trained in one condition consistently predicted the opposite when tested on the other condition."

R1.9 Please mention under the participants heading that three additional participants were excluded for source analysis due to unavailable MRI's.

Response: Done

R1.10 Why did you use logistic regression for decoding rather than the more common linear regression?

Response: We used logistic regression because our predicted variable was categorical (mel1 vs mel2) amounting to binary classification.

Reviewer #2:

Dear Dr. Schnell,

Dear Authors,

I thoroughly enjoyed reviewing "Decoding reveals the neural representation of perceived and imagined musical thoughts" submitted for publication in "PLOS Biology" (review request received: 27 June 2024, review accepted: 01 July 2024, review completed: 08 July 2024). This research opens a new door in understanding the perceived and imagined melodies from MEG data and is novel in its contribution to the current field researching imagination, music, and the brain. Particularly, MVPA analysis adds a new flavour to this investigation, which contributes to other neuroimaging techniques being employed, specifically studying the auditory cortex. In general, the work is very well written, is effective in conveying the information directly to the reader and fits within the scope of "PLOS Biology", and I recommend this manuscript for publication as it presents a valuable impact to the field. It is already in great shape; however, I have some comments that I recommend the authors should address in revision. I intend these comments as constructive feedback to support and enhance your efforts.

Comments

Abstract

R2.1 Comment: "With little effort you could sing it or play it vividly in your mind." I find this sentence too generalised. While many people can do this, this ability may not come naturally to everyone. I guess with could, you tried to convey this, however, I believe rephrasing it may enhance clarity.

Response: We have made the sentence more nuanced: "Vividly imagining a song or a melody is a skill that many people accomplish with relatively little effort."

Introduction

R2.2 Comment: The "Happy birthday" example is very commonly used. It is effective, but reference(s) are needed here, e.g.,

Cotter, K. N., Christensen, A. P., & Silvia, P. J. (2019). Understanding inner music: A dimensional approach to musical imagery. Psychology of Aesthetics, Creativity, and the Arts, 13(4), 489. Küssner, M. B., Taruffi, L., & Floridou, G. A. (Eds.). (2023). Music and Mental Imagery. London and New York: Routledge.

Halpern, A. R. (2012). Dynamic aspects of musical imagery. Annals of the New York Academy of Sciences, 1252(1), 200-205.

Poliakoff, E., Bek, J., Phillips, M., Young, W. R., & Rose, D. C. (2023). Vividness and Use of Imagery Related to Music and Movement in People with Parkinson's: A Mixed-methods Survey Study. Music & Science, 6, 20592043231197919.

Di Liberto, G. M., Marion, G., & Shamma, S. A. (2021). Accurate decoding of imagined and heard melodies. Frontiers in Neuroscience, 15, 673401.

Response: Thanks a lot for pointing us towards this literature. We have included these references in the relevant places.

R2.3 Comment: p. 13, fMRI and EEG to be spelled out the first time.

Response: Done

R2.4 Comment: Introduction lacks literature that is current and related to this study, such as auditory imagery with or without MEG. For instance, very relevant works include:

Herff, S. A., Herff, C., Milne, A. J., Johnson, G. D., Shih, J. J., & Krusienski, D. J. (2020). Prefrontal High Gamma in ECoG tags periodicity of musical rhythms in perception and imagination. Eneuro, 7(4).

Dash, D., Ferrari, P., & Wang, J. (2020). Decoding imagined and spoken phrases from non-invasive neural (MEG) signals. Frontiers in neuroscience, 14, 290.

Cheng, T. H. Z., Creel, S. C., & Iversen, J. R. (2022). How do you feel the rhythm: Dynamic motor-auditory interactions are involved in the imagination of hierarchical timing. Journal of Neuroscience, 42(3), 500-512.

Schaefer, R. S. (2014). Images of time: Temporal aspects of auditory and movement imagination. Frontiers in Psychology, 5, 877.

Dijkstra, N., Kok, P., & Fleming, S. M. (2022). Perceptual reality monitoring: Neural mechanisms dissociating imagination from reality. Neuroscience & Biobehavioral Reviews, 135, 104557.

Dijkstra, N., & Fleming, S. M. (2023). Subjective signal strength distinguishes reality from imagination. Nature Communications, 14(1), 1627.

Response: We have included these citations in the relevant places.

R2.5 Intro can also benefit from more primary music-related imagery work that is associated with your work, as there are many imagery modalities - auditory, vision -, and this body of literature below, for instance, is currently investigating specific/overall modalities. It is worth pointing out here what imagery modalities you exactly are investigating. If that is not modality-specific, that is also okay, but

I cannot be exactly sure whether it is auditory or other modalities as well that you are investigating here from the text, or the definition of the "imagination" is not too clear.

Küssner, M. B., & Taruffi, L. (2022). Modalities and causal routes in music-induced mental imagery. Trends in Cognitive Sciences.

Taruffi, L., Ayyildiz, C., & Herff, S. A. (2023). Thematic Contents of Mental Imagery are Shaped by Concurrent Task-Irrelevant Music. Imagination, Cognition and Personality, 43(2), 169-192.

Herff, S. A., Cecchetti, G., Taruffi, L., & Déguernel, K. (2021). Music influences vividness and content of imagined journeys in a directed visual imagery task. Scientific Reports, 11(1), 15990.

Herff, S. A., McConnell, S., Ji, J. L., & Prince, J. B. (2022). Eye Closure Interacts with Music to Influence Vividness and Content of Directed Imagery. Music & Science, 5, 20592043221142711.

Margulis, E. H., & Jakubowski, K. (2024). Music, Memory, and Imagination. Current Directions in Psychological Science, 33(2), 108-113.

Margulis, E. H., & McAuley, J. D. (2022). Mechanisms and individual differences in music-evoked imaginings. Trends in Cognitive Sciences.

Response: Thanks for the input. We have made it clearer in the introduction and the abstract that we study here auditory imagery, particularly for the case of musical sounds. We also mention the cross-modal studies you refer to in the last paragraph of the discussion:

> "*In conclusion, our results provide evidence regarding the nature and dynamics of perceived and imagined sound representations in the brain and contribute to a growing body of work investigating musical imagery and its relationship with other modalities (2,58–63).*"

R2.6 Comment: "However, no study has elucidated 1) the nature of sound sequence representations in auditory and association areas, 2) how these representations evolve over time and 3) how they change when mentally manipulated." - "no study" is simply untrue and very argumentative within this sentence as there are many studies looking at these aims (such as above and some of the work you've already cited). Although your study is novel in its aims noted elsewhere, please change the wording here or change the sentence in a way that no study studied what you exactly are looking at here.

Response: We have changed the phrase to convey more nuance:

> "However, it remains unclear **1)** how sound sequences are represented in auditory and association areas, **2)** how these representations evolve over time, and **3)** how they change when mentally manipulated."

Results

R2.7 Comment: "Participants (N = 71, 44 female, age = 28.77 +/- 8.43 SD) performed with high accuracy (Fig. 1c) and were better (OR = 1.85, CI = [1.25 - 2.72], p = .002) in the recall (96.7%, CI = [95.6 - 97.6]) than the manipulation (94.1%, CI = [91.9 - 95.8]) block. This confirms the presence of melody specific information during the imagination period." - This finding can also be interpreted in other ways, such as differences in task difficulty or participant engagement between the recall and manipulation blocks that might be crucial to understand the differences of the recall and manipulation blocks.

Feel free to ignore this, but maybe, it is worth pointing out in the discussion.

Response: Indeed, there was a difference between conditions regarding task difficulty and this was expected based on previous research. However, given the high accuracy achieved in both blocks, the subtle performance differences do not affect the conclusion that information about the melodies was present in both conditions.

R2.8 Comment: "This is indicative of a flip in neural representations such that models trained in one condition were consistently "wrong" when tested on the other condition." - Here, I feel "wrong" is the wrong word to use here. Possibly, "inaccurate" might be a better word as it is more precise and scientifically appropriate in the context of neural representations and model testing.

Response: In response to reviewer 1 (R1.8), we have changed this whole paragraph to explain better the logic of between-condition testing. We hope the writing is clearer now:

> *"This indicates a flip in neural representations such that models trained in one condition consistently predicted the opposite when tested on the other condition."*

R2.9 Comment: Sound and tones are interchangeably used. It is worth keeping these terms consistent.

Response: Done

Discussion

R2.10 Comment: 2nd and 3rd paragraph of the discussion could be combined as the 2nd paragraph feels incomplete, and they seem to be very short paragraphs. There are missing references too, and it is unclear whether the last sentences are speculation or not to the general reader, so references would go a long way here.

Response: We have combined the paragraphs. The last two sentences are our own interpretation. We have rephrased slightly to make this clearer:

> *"During listening, this could be due to the constantly changing sensory input. During imagination, this might reflect temporal variability between participants."*

R2.11 Comment: "Temporal order is an abstract feature that might generalize less across listening and imagination than sensory features such as pitch, which are typically the target of decoding."- missing reference here. E.g., Sankaran, N., Thompson, W. F., Carlile, S., & Carlson, T. A. (2018). Decoding the dynamic representation of musical pitch from human brain activity. Scientific reports, 8(1), 839.

Response: We have included this reference. Thank you.

R2.12 Comment: I'd also include references to the omission studies examples as well as for the fMRI ones.

Response: Done.

R2.13 Comment: "...which might introduce between-trial and between-subject variations that blur sound-specific representations.". - I think it could be important to highlight how this could be addressed, such as including more trials to enhance the reliability of the data, or considering using mixed-effects models to account for both fixed (conditions) and random (participant variability). I

guess running mixed-effects models could address this limitation most straightforwardly within this paper, but that is up to the authors' choice, as simply addressing the limitation and how to address it in the future is also okay.

Response: At the end of the discussion, we explicitly mention how this limitation could be addressed:

> *"Future work might use different recording modalities (e.g., optical MEG, intracranial EEG), bigger datasets (e.g., by increasing the number of trials), and models that are larger and account for the temporal variability in imagination (e.g., deep learning) (64) to maximize the decoding of auditory images."*

Regarding mixed effects analyses, a decoding model including participant as random effect would pose unique challenges, as the neural patterns might not be sufficiently aligned across sensors between subjects and the number of subject-specific random effects would be too large. This is an interesting idea that we would like to explore in future work.

R2.14 Comment: "previous neuroimaging activation studies as important for auditory imagery." - Is this only relevant for auditory imagery or other modalities too? Or is there something special related to auditory imagery here?

Response: Indeed, these areas are important for both audition and other modalities. We have rephrased to reflect this:

> *"The regions that carried auditory representations in our study overlap with those identified in previous neuroimaging activation studies as important for imagery in audition and other modalities."*

R2.15 Comment: "Furthermore, we observed melody specific representations in the basal nuclei, a set of areas involved in both cognitive and motor control that have not been identified in previous fMRI research" - Here, I find "previous fMRI research" very specific. It might be worth clarifying which fMRI research, such as including "... fMRI research on melody processing." And I might be missing something here, but why only fMRI research? How about EEG, etc., or is this only an example?

Response: The emphasis on fMRI is because it has been so far the most reliable method to measure activity in deep brain structures. EEG does not have enough spatial resolution to do that. We have deleted the reference to fMRI to avoid confusion:

> *"...a set of areas involved in both cognitive and motor control that have not been identified in previous auditory imagery research."*

R2.16 Comment: "Finally, our results demonstrate the feasibility...". - I would change "Finally" to "Overall" as finally read like it is the final point you want to make. This paragraph could take advantage of adding a sentence regarding the purpose of the study and how this demonstrated in your results.

Response: We have changed the opening phrase to make the paragraph more conclusive:

> *"In conclusion, our results provide evidence regarding the nature and dynamics of perceived and imagined sound representations in the brain..."*

Method

R2.17 Comment: For stimuli, no need for "timbre" for piano, as this can be confusing to the general reader if the definition of timbre is not being used before (as it is a complex subject and definition is ill-defined and in debate (Siedenburg et al., 2019), just leaving "piano" would be sufficient.

Response: Done

R2.18 Comment: The stimuli section is too brief. It lacks information on whether the stimuli are recorded (if it was, how?), or taken from elsewhere, or produced/ artificially made? Did the piano sound natural or not (e.g., artificially made)? Currently, one cannot replicate re-producing the stimuli.

Response: We have added details in the methods section:

> *"We employed short three-note melodies forming a major chord arpeggio using piano sounds (musical pitch: A3, C#5, E6; F0: 220Hz, 554Hz, 1318Hz) synthesized with MuseScore (v3.6.2; see materials' GitHub repository for the actual sounds used)."*

Additionally, now we point the reader to our materials' GitHub repository where the actual sounds used can be found:

https://github.com/drqm/memory_music_iEEG/tree/master/stimuli/manipulation_normalized

R2.19 Comment: melody1 and melody2 are written as melody 1 and melody 2 at different points of the Method section.

Response: We have made sure that all the instances were written equally.

R2.20 Comment: Regarding the task, how long did the experiment take?

Response: Approx. 20 minutes. We have added this to the methods section:

> *"Sound stimulation was delivered through magnetically isolated ear tubes. The task lasted approximately 20 minutes. Other experimental paradigms testing recognition memory were recorded together with this task."*

R2.21 Comment: "using the inverse solutions described above (Fig. 1f)"- in Figure 1f, instead of above.

Response: Actually, what we meant was: "the inverse solutions described in section 3.5. (**Fig. 1f**)"

R2.22 Comment: For completeness, I liked the addition of relationship with behaviour. Is there a missing citation here: "(R, lme4; 52, 53)? However, it might also be worth adding this section to the supplementary materials instead of here as this does not significantly add to the main points. Words could be used more to improve the Introduction and Discussion section.

Response: No reference is missing; we had a typo. We have moved this section to the supplement and have added some other correlation analyses between neural decoding and music training and vividness scores (see response to comment **R1.6** for details).

R2.23 Comma Comments:

- Oxford comma inconsistency
- Missing commas (e.g., "Interestingly, after the second sound, melodies were discriminated in association, sensorimotor and subcortical structures ($p < .005$), but not in auditory areas (Fig. 3b).

Response: We have fixed the use of Oxford comma wherever needed.

R2.24 Thank you a lot for your fascinating contribution, and I look forward to seeing a revised and published version of this study.

Response: Thank you for your valuable input!

Reviewer #3: This is a well conducted study that provides a valuable contribution to the literature. I have only the following brief comments.

R3.1 It is not clear what is meant with "This confirms the presence of melody specific information during the imagination period". How is performing accurately conclusive evidence of imagination during the imagination period? As per above, I would suggest that "This further confirms that mental representations were present during the delay period." be amended to "This confirms that mental representations were present during the delay period"

Response: We agree with the reviewer that this is not conclusive evidence and have changed the word "confirms" to "suggest". We also acknowledge this limitation in the discussion. That said, we note that our between-condition decoding results provide evidence that participants actively engaged with the mental recall and manipulation of sound sequences. Furthermore, we now report subjective vividness ratings that the participant gave after completion of the task indicating that most of them experienced mild-to-strong imagery vividness (see also response to R1.2):

> *"After the experiment, participants rated task-related imagery vividness on a 7-point Likert scale (from -3 to 3), with 72% of them rating 0 or above, which ranges from mild to strong vividness (Table 1). The good task performance and the vividness ratings suggest the presence of melody-specific information during imagination."*

Table 1. *Vividness ratings at the end of the task across participants.*

**Rating**	**-3**	**-2**	**-1**	**0**	**1**	**2**	**3**
**Number of participants**	1	4	8	10	25	12	4
**Percentage**	1%	6%	11%	14%	35%	17%	6%

R3.2 Also note that neither "imagination period" or "delay period" are introduced clearly. Presumably they are the same? Introduce one and stick to it.

Response: We kept "imagination" and changed the wording in the relevant places.

R3.3 In "We also considered the possibility that representational dynamics were different between recall and manipulation. This is interesting because, during manipulation, the representation of the listened melody is meant to be the opposite of the imagined melody, potentially yielding different temporal generalization patterns.", make clear WHAT is interesting. The possibility is interesting?

Response: We have deleted this phrase because the relevance of this result is clear from the rest of the paragraph.

R3.4 This is not clear" The resulting activity patterns can be interpreted as the differences between melodies that underlie successful decoding". Rephrase, or just delete since the line before seems adequate.

Response: This is an important detail. We have rephrased for clarity: *"The resulting patterns can be interpreted as the differences in neural activity that discriminate between melodies and underlie successful decoding."*

R3.5 In "The same areas represented the melodies after the third sound ($p \leq .021$; Fig. 3c)", make clear whether you mean as for the first or second sound.

Response: Done. "*The same areas outlined above represented the melodies after the third sound.*"

R3.6 Elaborate on this statement" Furthermore, the detection of deep sources has become increasingly common, including the basal nuclei, the medial temporal lobe and even the cerebellum (43-48)". Why? Better methods?

Response: The use of beamformers (with the appropriate controls) has made the detection of deep sources possible. We rephrased accordingly:

> *"Furthermore, with implementation of appropriate controls, the use of beamformers has made the detection of deep sources increasingly common, including the basal nuclei, the medial temporal lobe, and even the cerebellum"*

References

1. J.-R. King, A. Gramfort, A. Schurger, L. Naccache, S. Dehaene, Two Distinct Dynamic Modes Subtend the Detection of Unexpected Sounds. *PLOS ONE* **9**, e85791 (2014).
